# Integrated Disease Surveillance and Response (IDSR) in Malawi: Implementation gaps and challenges for timely alert

**DOI:** 10.1371/journal.pone.0200858

**Published:** 2018-11-29

**Authors:** Tsung-Shu Joseph Wu, Matthew Kagoli, Jens Johan Kaasbøll, Gunnar Aksel Bjune

**Affiliations:** 1 Department of Informatics, University of Oslo, Oslo City, Norway; 2 Research Department, Luke International, Mzuzu City, Malawi; 3 Overseas Mission Department, Pingtung Christian Hospital, Pingtung County, Taiwan; 4 Department of Public Health, National Taiwan University, Taipei City, Taiwan; 5 Department of Epidemiology, Ministry of Health, Lilongwe City, Malawi; 6 Public Health Institute of Malawi, Lilongwe, Malawi; 7 Institute of Health and Society, University of Oslo, Oslo City, Norway; The University of Warwick, UNITED KINGDOM

## Abstract

**Objective:**

The recent 2014 Ebola Virus Disease (EVD) outbreaks rang the bell to call upon global efforts to assist resource-constrained countries to strengthen public health surveillance system for early response. Malawi adopted the Integrated Disease Surveillance and Response (IDSR) strategy to develop its national surveillance system since 2002 and revised its guideline to fulfill the International Health Regulation (IHR) requirements in 2014. This study aimed to understand the state of IDSR implementation and differences between guideline and practice for future disease surveillance system strengthening.

**Methods:**

This was a mixed-method research study. Quantitative data were to analyze completeness and timeliness of surveillance system performance from national District Health Information System 2 (DHIS2) during October 2014 to September 2016. Qualitative data were collected through interviews with 29 frontline health service providers from the selected district and 7 key informants of the IDSR system implementation and administration at district and national levels.

**Findings:**

The current IDSR system showed relatively good completeness (73.1%) but poor timeliness (40.2%) of total expected monthly reports nationwide and zero weekly reports during the study period. Major implementation gaps were lack of weekly report and trainings. The challenges of IDSR implementation revealed through qualitative data included case identification, compiling reports for timely submission and inadequate resources.

**Conclusions:**

The differences between IDSR technical guideline and actual practice were huge. The developing information technology infrastructure in Malawi and emerging mobile health (mHealth) technology can be opportunities for the country to overcome these challenges and improve surveillance system to have better timeliness for the outbreaks and unusual events detection.

## Introduction

After the deadly Ebola Viral Disease (EVD) outbreak in Western Africa, governments, health authorities in Africa and the world learnt valuable lessons from the challenges of diseases surveillance systems implementations in countries with limited public health infrastructure [1, 2]. The outbreak emerged in 2013, ended in June 2016 and affecting 10 countries with 11,310 deaths and 28,616 cases (including confirmed or suspect). [3–6]. The fragility of the public health infrastructure and capabilities, to capture early warning signal of outbreak and provide good timeliness for response was further exposed during this outbreak. Calls for action highlighted the need for strengthening the surveillance system in these countries and transform it from passive to active surveillance [2, 7]. Despite the efforts, new EVD outbreak emerged in the Democratic Republic of the Congo in April 2018 [8].

Early case detection is one of the important approaches to managing future outbreaks [9]. Although Integrated Diseases Surveillance and Response (IDSR) strategy was adopted in most countries in Africa since 1998, still, challenges of implementing IDSR was evident even before the tragic EVD outbreak in 2014 [10–12]. Following the Severe Acute Respiratory Syndrome (SARS) outbreak in 2003, the International Health Regulation (IHR) was revised by the World Health Organization (WHO) in 2005 and fully adopted by most countries around the world [13]. The IHR-2005 enhancement proved to be helpful in dealing with the 2009 H1N1 influenza pandemic and IDSR strategy serves the platform for its implementation in Africa [14, 15]. However, shortcomings of the global health system’s capabilities, lack of virological surveillance in Africa and technologies for vaccine production and implementation and the basic public health system infrastructure were exposed during the same pandemic [16].

Malawi adopted the IDSR strategy in 2002 and the third edition technical guidelines were published in May 2014 with incremental notifiable diseases and health conditions to fulfill the IHR-2005 and public health needs [17]. Despite existing framework of IDSR system, few nationwide assessments of IDSR system have been done in Africa and none in Malawi [12, 18–20]. This study aims to describe the process of case identification and reporting in practice, and explore the differences between the IDSR guideline and actual implementation using timeliness and completeness as key indicators to evaluate IDSR performance in Malawi.

## Materials and methods

### Study setting

**Fig 1:**
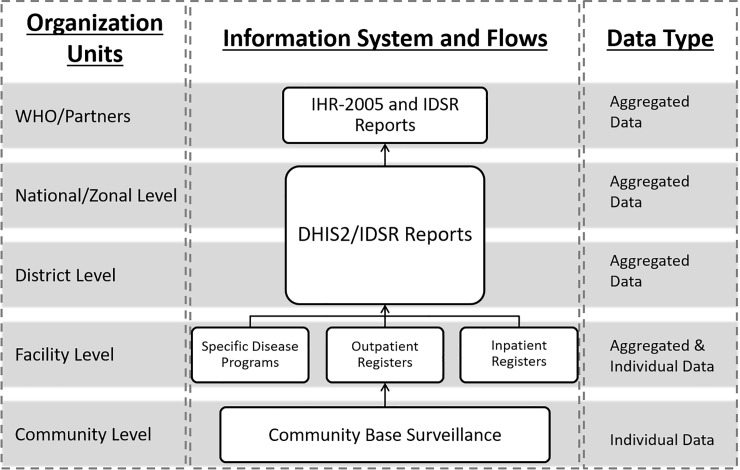
The IDSR system information flow according to the organization architecture in Malawi.

Malawi is a land-locked country situated in south-eastern Africa with a population of almost 16 million. It is bordering with Tanzania, Zambia and Mozambique [21]. Administratively, the country is divided into 3 regions, northern, central and southern region, with further demarcation into 5 zones, 29 health districts and 4 major cities. The official language of Malawi is English and national language is Chewa. Tribal languages are used for communication in different districts, including Nyanja, Tumbuka, Tonga, Yao, etc [22]. The epidemiology department (ED) of the Ministry of Health (MOH) is the main custodian of the IDSR system while the Center for Central Monitoring and Evaluation Division (CMED) in the Department of Planning and Policy Development in the MOH is responsible for coordinating the routine Health Management Information System (HMIS) and its subsystems, including IDSR [23]. The IDSR system reporting and information flow follows the health system organization structure from the community to the national level (Fig 1).

At the community level, the Health Surveillance Assistants (HSAs) are the frontline health care workers (HCWs) responsible for case identification and report. They work under the supervision of attached health facilities to identify case and further refer to the nearest health facility [24]. Out of 1,060 health facilities in Malawi, 64% are public government owned or under the Christian Health Association of Malawi (CHAM). The remaining 36% are private for-profit or non-governmental organizations facilities under government regulatory [25]. The HCWs at each facility, irrespective of ownership (public or private) are responsible for case identification and reporting (weekly and monthly). Each facility has a person responsible for tallying reportable cases using various health information tools, including electronic medical records (EMR) system [26]. In the current guideline, 19 diseases and conditions are required immediately reporting (S1 Table).

Each District Health Office (DHO) has a District Health Management Team (DHMT) overseeing health programmes. The District Environmental Health Officer (DEHO) of DHMT is responsible for HSAs management and the district IDSR focal person is collecting surveillance reports from facilities for submission and notification. From district level above, Malawi has adopted District Health Information System (DHIS) as the national system for HMIS reporting since 2002. The system was upgraded to a web-based open-source information system, DHIS2, in 2012 [27]. MOH is hosting DHIS2 and the IDSR datasets are required to be entered intpDHIS2 by the focal person since late 2014. The reported IDSR data is stored in a centralized server managing by CMED. The IDSR system’s core functions at each levels of the health system are clearly articulated in the guideline including identify, report, analyze and interpret, and investigate and confirm [17].

### Study design

The study mixed quantitative and qualitative methods to assess and understand the implementation gaps of IDSR system in Malawi [28]. The study focused on two key attributes, timeliness and completeness, of the surveillance system for quantitative analysis [29]. The study adopted two main core functions of IDSR guideline, case identification and reporting, as the key themes for further qualitative analysis.

### Source of quantitative data

We used the built-in function of the DHIS2, Reporting Rate Summary, to extract IDSR monthly reporting rate summary data from the central server of the Ministry of Health. The studied period was from October 2014 to September 2016 and the data were extracted by district in June 2017. The reporting rate summaries function provides the completeness and timeliness indicators for analysis. Completeness of reporting for the district was calculated using numbers of actual reports received against numbers of expected reports from health facilities at DHO. Completeness is represented in percentage format and used as a quality indicator to monitor the performance of IDSR system [17]. Another way the DHIS2 can calculate completeness is to monitor whether the compulsory data elements were entered or not. However, such way of completeness calculation was not included in this study scope. Timeliness of reporting was calculated based on the proportion of health facilities submitting surveillance reports on time to the district. According to the national policy, the facility IDSR monthly report is considered “on time” if it was entered into the DHIS2 system before the last day of the following month. The IDSR technical guidelines sets the district performance target at 80% for timeliness.

### Source of qualitative data

Qualitative data were collected through key informant interviews and focus groups from one selected district in the Northern Region of Malawi, which had the best performance in terms of completeness of IDSR reporting in 2013 (S2 Table). Key informants included health workers involved in IDSR duties from the community to district level. Since the IDSR is a national program and all districts are implementing with the common standard across the country, the MOH intends to strengthen the system with a nationwide approach that may lead to general improvement of the surveillance system. With the government intention in mind, the researchers chose the best performing district to see whether it will be at all realistic to use the district experience for IDSR strategy implementation improvement. The assumption was that the challenges identified in the best performing district are the key barriers for success of the nation-wide IDSR implementation. The practices done in the study district can also create ideas for further research to identify success factors for achieving good completeness and remaining challenges that contribute to poor timeliness. We assumed the districts with poor performance (completeness) may have a multitude of challenges with its own specific contexts.

The male researcher (TSJW) conducted the interviews and observations based on the interview guide in English, Chewa or Tumbuka (S1–S3 Files). The researcher (TSJW) resides in Malawi and has been working with the MOH for health system strengthening since 2007. Despite the researcher’s ability to use the primary local language, he understands the HSAs are mainly using Tumbuka for communication in the studied district. To avoid communication difficulties with the HSAs, the researcher engaged a local female trained research assistant to facilitate interviews. In order to identify the HSAs within the district, the researcher engaged the DHO to accompany the research team in the field. At the community level, the research team visited 17 HSAs who run village clinic. At the facility and district level, the team planned to conduct focus group discussions with HCWs and DHMT with 10 and 5 proposed participants respectively. The researcher observed operation of the outpatient clinic in hospitals to obtain contextual information about the service provision and IDSR report generating process in the study district. All interviews and observations were administered after obtaining consents from the interviewees and digitally recorded for transcribing and translating by the local research assistant. The filed study was conducted during August and September in 2016. For the leading positions at the national level, the researcher relied on interactions with key informants from the ED and CMED to gain surveillance related implementation experiences and challenges. Upon consent from the key informants, meeting notes were taken during these interactions for further analysis. Saturation of data was considered as a stop signal when no additional information were provided by additional interviewees.

### Ethics approval and consent to participate

The study protocol was reviewed and approved by the National Health Sciences Research Committee (NHSRC) of Malawi with approval number 16/4/1563. The study was granted permission by the health authorities from district health office and the Ministry of Health. All interviews were conducted with the written consent from the interviewees.

### Data analysis

Quantitative data were extracted from the national DHIS2 instance and exported into Microsoft Excel for further analysis. Line charts were used to illustrate the time series patterns of the IDSR monthly report data quality. The differences of completeness and timeliness between zones/districts and national average were compared using one-tailed proportion Z-test. The data were further stratified by dry season (May to October) and rainy season (November to April) and tested using two-tailed proportion Z-test to compare seasonality differences at each levels of health system. This was taking consideration the hypothesis of transports challenges might be affected by the climate [12]. The level of significance was set at α = 0.05. The timeliness data were compared against the performance target (80%) in each district.

Qualitative data were imported to NVivo 12 software for coding and analysis. The researcher used thematic analysis approach to categorize data according to the two key themes from the IDSR core functions–identification and reporting. A priori codes were developed based on the identification and reporting themes to guide deductive analysis. The core functions of each level of health system actors were compared with the expected functions according to the guideline. Empirical codes were developed inductively during the analysis to identify concepts from the data relating to implementation challenges on case identification and reporting, especially themes may relate to timeliness.

## Results

### Quantitative data

**Fig 2:**
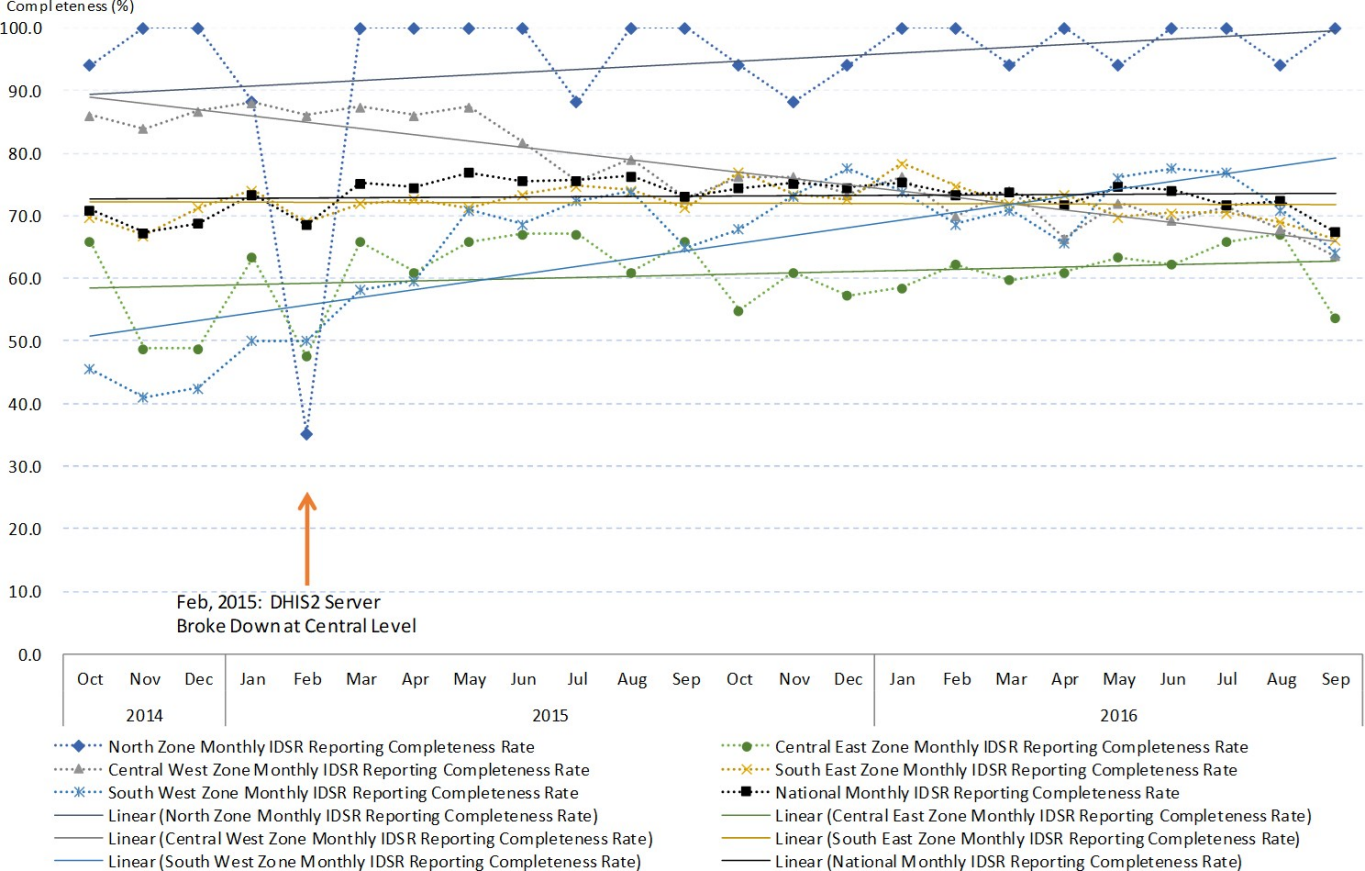
The IDSR monthly reports completeness indicator from October 2014 to September 2016 divided by zones and national level.

**Fig 3:**
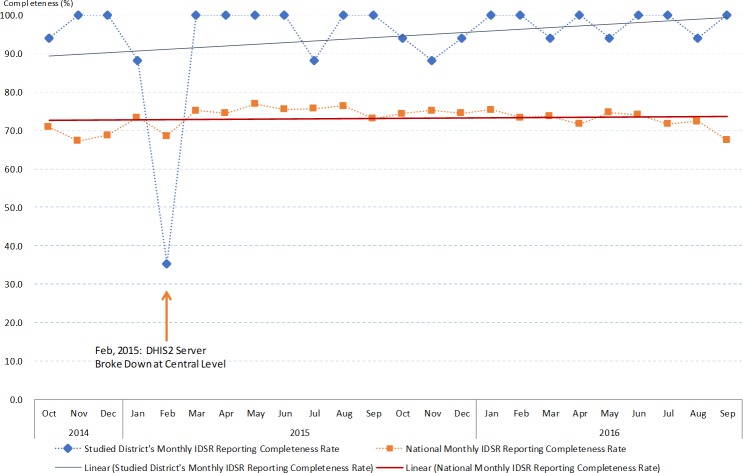
The IDSR monthly reports completeness indicator from October 2014 to September 2016 at the studied district and national level.

We extracted 648 IDSR reporting rate summary data from DHIS2 during the 24 months study period of all health districts in Malawi (S5 File). The completeness data showed national average completeness was 73.1% and 94.4% in the studied district. Nationwide difference of completeness between dry and rainy season was not significant. However, significant seasonaility variation was observed in some districts, including the studied district (S3 Table). The time series pattern for monthly completeness showed trends of improving in four out of the five health zones. The same was also observed in the studied distrct (Figs 2 and 3). In the studied district, the completeness performance remained significantly better than the national average (S3 Table).

**Fig 4:**
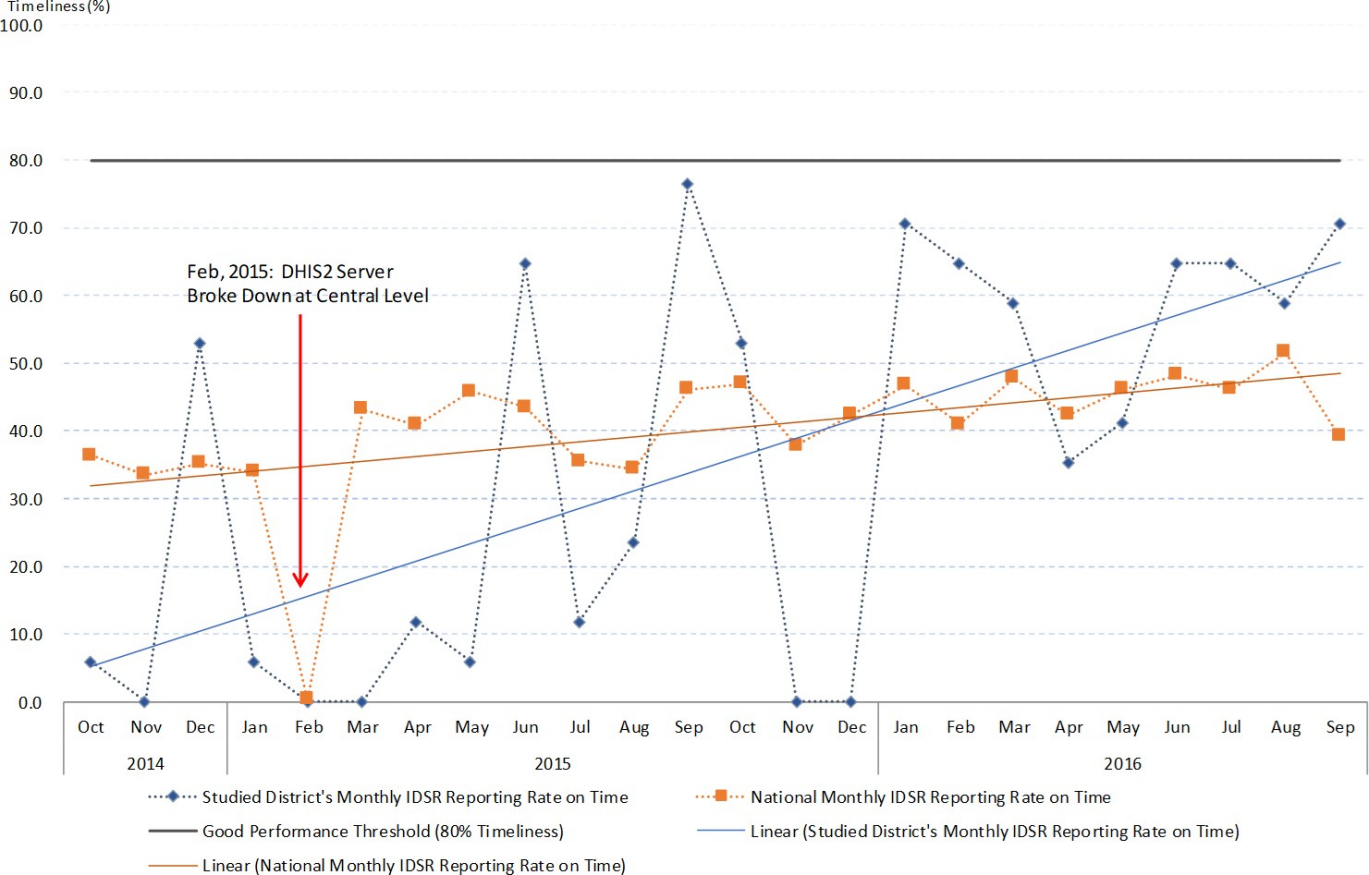
The IDSR monthly reports timeliness indicator from October 2014 to September 2016 at the studied district and national level.

**Fig 5:**
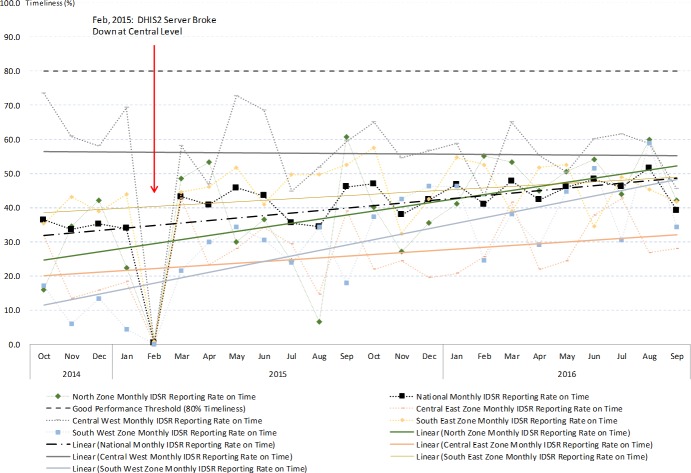
The IDSR monthly reports timeliness indicator from October 2014 to September 2016 divided by zones and national level.

The IDSR monthly report showed very poor performance in terms of timeliness. On annual basis, very few districts reached the target of 80% on-time reporting and the study district did not achieve it throughout the study period (Fig 4). Seasonality differences were observed significantly at the national level, as well as in four health zones and the study district (S4 Table). Timeliness was significantly poorer in the study district compared to the national average (S4 Table). However, the time series pattern for monthly timeliness showed trends of improving in four out of the five health zones, as well as in the study district (Figs 4 and 5). Notably in February 2015, the timeliness of IDSR monthly reports all dropped to 0% due to server breakdown. This also affected the completeness of IDSR monthly reports throughout the country. These findings indicate that the study district maintained good IDSR system performance from the completeness aspect, but challenges exist that affect performance on timeliness of reporting. The challenges encountered and the practice of IDSR on the ground level are further explored by thematic areas using data collected through the qualitative study arm.

### Qualitative data

Upon the data collection, 17 HSAs were approached at the community level while 2 of them did not provide sufficient information to the study. Twelve HCWs participated in two focus group discussions from two health facilities in the studied district. At the district level, four officers from the DHMT participated in the study. Three key informants were approached and provided IDSR related information through interactive meetings and interviews at the Ministry of Health departments. As a result, we included qualitative data from 34 informants for analysis and data reached saturation as no further information was provided in related to the IDSR implementation and challenges. Majority of the informants were male (N = 22) age between 28 to 52 years old. Field notes were taken by the researcher (TSJW) during actual clinical practice and operation of IDSR observations.

All qualitative data were coded with the 77 concepts according to a-priori and emerging concepts. We identified key themes and sub-themes as Table 1. Based on the analysis result, we summarized the finding of comparison between IDSR technical guideline and actual practice of two studied surveillance core functions as S5 Table.

**Table 1:** Key themes and sub-themes identified from key informants.

Key themes	Sub-themes
● Case identifications	● Case definitions ● Actual case identification practice ● Tools for surveillance ● Lab confirmation
● Reporting	● Generating reports ● Modes of reporting
● Implementation gaps	● Weekly IDSR reports ● Trainings and supervision
● Challenges	● Case identification ● Compiling report for timely submission ● Financial, capacity and infrastructure

### Case identification

#### Case definition

At the community level, according to all the informants, none of them was practicing community-level case identification using IDSR guidelines. They were familiar with the case definitions when it comes to child illness based on the integrated community case management (CCM) guideline.

*“We have to observe how the baby is breathing by counting bullets per minute using a watch*. *We have specific guidelines* (CCM) *for children one year and below…”* HSA, #RU03

Even at the facility level, HCWs were not only using IDSR guideline as the major case definition reference but also other clinical guidelines.

*“There are guidelines for malaria… and guidelines for ARI…*. *So it depends on the presentation of the patient*, *as you are going for assessment*, *and then you compare which guideline tallies the presenting symptoms*.*”* HCW, #RDH03

The unaligned implementation of case definitions using different guidelines may affect the accuracy of the surveillance data and incomplete information.

*“We are still using the old* (IDSR) *guidelines which don’t have enough information while the new* (IDSR) *guidelines have got more information so we are just waiting for the orientation*.*”* DHMT, #RDHO02

#### Actual case identification practice

We further analyzed how HSAs and HCWs performing actual case identifications at community and facility level. According to the IDSR technical guidelines, the HSAs are supposed to do active case finding to identify priority diseases and public health events. Instead of going to the villages, they relied on volunteers from the Village Health Committee (VHC) to report unusual health events. The health volunteers from the VHC hence played critical roles for the community level outbreak or incidence alerts.

*“I have volunteers from each village*, *2 of them* (in each village). *Those volunteers are my ambassadors*. *They have the knowledge*, *if any outbreak*, *they tip me*, *then I rush* (to the village).*”* HSA, #RU03

Apart from the helps of VHC, the HSAs identify notifiable cases through operating the village clinic. The clinic was designed mainly to provide health services to under five children, but the adults are also access the clinic to get consultations. Upon case detected, the HSAs referred both children and adult cases to the next level health facilities.

*“Sometimes I may find children with various diseases some of which I cannot treat and I refer such cases to the hospital*.*”* HSA, #MH04

*“After counselling them*, … *I then make a referral to the health facility for further assessment which will be done at the health facility”* HSA, #MH01

The main function that HSAs saw for themselves in serving IDSR was to assist their attached health facilities to compile the IDSR monthly reports, perform community sensitization and education. Through observations, some HSAs were equipped and capable to do simple data analysis and use them as instrument to interact with the VHC for disease prevention and health promotion. However, they did not perform active case finding based on the community case definitions as guideline suggested.

*“At first*, *we are going to each and every household…but this time it’s not often*.*”* HSA, #RU03

The facility level HCWs identify cases based on their clinical judgements and epidemiological history. These identified cases were recorded in various paper registers or computer system for surveillance purposes.

#### Tools for surveillance

At the community level, the HSAs use the CCM register and reporting form as the tool for collecting surveillance data. However, there is no standardized tool has been used to collect detected and referred cases from community. Another important tool that both HSAs and HCWs used for surveillance is outpatient clinic paper registers. In some facilities where computers are available, the IDSR focal person also used computer to combine with paper registers for surveillance data capturing.

*“So I may simply go to the register and capture the date and combine the data from the register with the one from the computer*.*”* DHMT, #RDHO02

#### Lab confirmation

Lab confirmation is an important activity of the case detection core function. The HSAs are performing malaria rapid diagnosis at the village clinic. When they encountered other priority diseases, i.e. tuberculosis, some of them collect specimen at community while others refer patients to the health facilities for further tests. This difference is depending on the specimen collection material’s availability.

*“When they come we ask them how many days have they been coughing*? *‘ah… it’s been 3 days now’*. *Then we give them bottles to take sample for TB test*.*”* HSA, #MH05

*“*(for TB) *you should go to the hospital to receive treatment*. *They are given small containers for collection of sample for TB test…*. *not in the community*.*”* HSA, #MH02

The management of cases was not based on the lab confirmation results. Most of the HCWs manage the patient based on the clinical judgements. But triggers for surveillance response were depending on the lab confirmation.

*“Actually we mainly do not have the diagnostic materials…yes…that’s why we just manage them clinically*.*”* HCW, #MHRH02

*“You are suspecting measles*, *then you take samples to the lab*, *and then they confirm it’s really measles*…. *You have to take the sample to the lab*, *and wait for confirmation*.*”* HCW, MHRH01

### Reporting

#### Generating reports

The HSAs generate reports using the CCM paper register and unstandardized reporting formats. The CCM reporting for surveillance was done on monthly basis while for the emerging events identified by community health volunteers were reported when the incident occurred. The HSAs gathered at the attached health facilities at the end of the month to generate reports using different registers.

*“At the end of every month*, *those HSAs residing in different catchment areas come to meet at the health centre*. *They meet to compile reports for the districts*. *So we also report during that time*.*”* HSA, #ML02

At the facility level, the same happened for the IDSR focal person to generate required monthly IDSR reports using various surveillance tools.

*“It is from the OPD registers where the focal persons in the health facilities also collect that data*. *So at the end of the month*, *they compile the data from the OPD registers and then they send it…the information from the children’s ward*, *… from the OPD registers*.*”* DHO, RDHO02

The HCWs picked up unusual health events through their daily services and did not wait until the monthly report to take actions.

*“When we see many patients are coming from that place and they are registering ARI* (acute respiratory infection). *What does that mean*? *So we don’t even wait for the month to come and work on the data*. *But we just see that I think for this… we come together and then we discuss*. *If it’s an outbreak we see that we cannot control*, *then we inform the DHO*.*”* HCW, #MHRH01

*“We usually report to the environmental officer and then they will send the HSAs and see what’s going on there*.*”* HCW, #RDH04

All facility generated IDSR monthly report then submitted to the DHO waiting for further submission.

#### Modes of reporting

The majority mode of reporting by HSAs was on paper based submission. We also discovered one HSA uses smartphone for nutrition program reporting but not in surveillance.

*“This one* (smartphone) *is for reporting I usually do reporting*. *I am a focal person of nutrition”*–HSA, #ML01

All paper reports from community and facilities were physically submit to the district health office, either through their own transport or wait for supervision teams to collect reports. These modes of report submission were depending on the availability of transports, supportive supervisions conducted by the DHO and road conditions during the rainy season. Apart from the regular monthly reports, they initiated preliminary investigations when community rumors emerge and physically walked to the higher-level health facilities to report.

*“I can write a written report then submit it to office*, *or I can go in person explain the situation to my boss*.*”* HSA, #RU02

At the district level, all reports were then entered into DHIS2 by the HMIS officers.

### Implementation gaps

#### Weekly IDSR reports

We noted during the field study at community, facility and district level that no one mentioned the IDSR weekly reports. It was observed that IDSR weekly reporting system was paralyzed due to the difficulties for HSAs and HCWs to cope with the overloaded catchment areas, volume of paper-generated reports and lack of internet connectivity. Each HSAs serves 3 to 24 villages and covering population size from 284 to 9,000 villagers. During the observation, a HCW provided 54 consultations within an hour and the person was not able to use computer system to capture any data at all. This seemed the main obstacle from national authorities’ perspective who eagers to enable the system for rapid responses.

*“Of course we told them to do weekly report*, *but there is no internet*. *For them to write report and send… it is just too difficult for them to handle these papers*.*”* ED, #MOH02

#### Trainings and supervision

Another major implementation gap was related to trainings and supervision. Lack of comprehensive training was an obstacle to enhance the electronic system to capture more data for disease surveillance and decision-making. It also affected the understanding of updated IDSR technical guidelines for HSAs and HCWS to do proper surveillance work.

*“The challenge is those who are using the computers*, *it’s just a few numbers of people who are oriented* … *that’s why it’s difficult to capture the data and many information*.*”* DHMT, #RDHO02

*“The new IDSR guidelines are in*, *but due to lack of funds they have not yet called us for orientation on the new guidelines*.*”* DHMT, #RDHO02

Supervision gap was huge and it directly affected the district performance on the quality of the IDSR tasks. According to the guideline, the MOH and DHO supposed to conduct regular technical supervisions to support filed surveillance works. Without proper supervision, the HSAs and HCWs were not able to get technical support and communication to improve their work performance.

*“for the past two or three years they used to do supervision*, *they used to see what is happening in the districts but for the past two years*, *they don’t do that anymore…We are supposed to do quarter preparations every year but for the past two years we did not make those official preparations”* DHO, #RDHO02

### Challenges

#### Case identification

Despite the understanding of most priority diseases case definition, the services provided at the community level and the health seeking behavior of community may affect the ability of HSAs to identify cases timely. The village clinic only provides services to under five children and lack of community based active case finding, the HSAs may miss out the adult cases and potential community outbreaks.

*“So only the under five children come here*. *I don’t deal with anyone older than that*.*”* HSA, #ML02

Traditional medicines were commonly use at the community level. Since the village clinic does not provide health services to adults, people would then use the traditional medicines prior to the formal health services. This affected the ability of formal health care system to identify cases in time.

*“Most adults first go to the traditional healer because when they come to us we can see they have tattoos on their chest cavity”* HCW, #MHRH01

*“And if we look at adults*, *especially women*. *When they have stomach pains or tumors*, *they first rush to the herbalist and usually come here when it’s way too late*. *They also come with marks on their body as treatment given by the herbalist*.*”* HCW, #RDH07

Another challenge related to case identification was lab diagnosis capacity. This also affected the ability of HCWs to identify notifiable cases.

*“Sometimes you find that maybe a patient may need chest x-ray…we don’t have it…sometimes it might happen that this patient needs full blood count in case of bacterial infections*. *We don’t do that either*.*”* HCW, #MHRH03

#### Compiling report for timely submission

There is a fundamental difference between the needs of the HMIS and the IDSR systems, where one is looking only for confirmed cases while IDSR is looking for alerts to take fast actions.

*“We want to get confirmed cases*. *We need to know exactly how many are they so we can do proper planning*. *That is why we want the data to be complete and accurate*.*”* CMED #MOH03

*“We need to know if there is something happen in the community*. *Wait for a month*, *sometimes three months to get report*, *it is just too slow*. *We need to take actions immediately so we are looking for any signal that can trigger us to take actions*.*”* ED, #MOH01

#### Therefore, the ED expected to use technology to improve timeliness and capability to get IDSR report automatically

*“If it is an immediately notifiable case*, *we want to know immediately*. *We don’t even want to wait them to report to us*, *we want to know now*. *Even it is a rumor or what*, *we need to know so we can check if it is true*. *That’s why we want to use this SMS or the eIDSR* (electronic IDSR) *so we can know there is something happening there*.*”* ED, #MOH02

However, despite of EMR system in place, the heavy workload made it difficult for HCWs to capture clinical information on system for automate report compiling. They simplified the work and transcribed individual level data into the different paper registers for surveillance use.

*“The computers are not fast as we expected them to be*. *Just to print somebody’s name*, *you have to wait for a minute or more*. *So you say this is delaying me*, *let me just write… we are having a lot of patients*. *A lot of them*.*”* HCW, #RDH04

The challenges to get timely reports through unstable information technology infrastructure were obstacles for the IDSR focal person in the DHO to provide quality reports.

*“…with IDSR*, *I have got challenges with the reporting system itself*, *from the health facilities*, *sometimes reports come a bit late*. *We also have challenges of that we do not have internet at the hospital*. *So we have to use the smart phones*, *the* (internet) *dongles to buy units and we are not provided with any funding for internet services so we have got to go into our pockets* …*”* DHO, #RDHO02

In the actual practice, the IDSR monthly reports were entered by the HMIS officer. The data quality of IDSR monthly report submitted through HMIS was the concern for ED to use. For instance, there were 31 Viral Hemorrhagic Fever cases recorded in DHIS2 in 2015, but none confirmed by the department.

*“If you look at the data*, *you will be surprised like*: *how can we have Ebola cases and we don’t know*. *The data quality is just so poor and we cannot use it*.*”* ED, #MOH01

#### Financial, capacity and infrastructure

Financial constrain was the root challenge for quality IDSR implementation. This created gaps for timely reporting, implementing weekly IDSR reports and having capacity to use the updated technical guideline at the community and facility levels.

*“We have to use our own money for us to access internet services as a result we end up delaying sending the reports which we have come up with*.*”* DHMT, #RDHO02

*“Otherwise you cannot explain everything in detail on the phone…*., *but it’s expensive*, *you need to have the money*.*”* HSA, #RU02

*“The guidelines are there only that they haven’t started implementing them yet because of maybe lack of funds and the like…we cannot just send them to the health facilities or HSAs without official orientation because it’s quite a big book”* DHMT, #RDHO02

Financial challenge also affected the laboratory diagnosis capacity and ability to use computer systems for surveillance data capturing.

*“It’s just a few people who are oriented which is a challenge*, *that’s why they find difficulties in capturing data”* DHMT, #RDHO01

The inadequate resources made the basic infrastructure of village clinics and health facilities insubstantial. Out of interviewed 17 HSAs, only one of them was working in a well-constructed building. Most of them were operating in an unfinished or substandard location, i.e. house without roof, under a big tree or the HSA’s own house, etc. where electricity was also scarce.

*“It’s very difficult and it’s a big problem*, *I haven’t seen anybody within Rumphi who has been allocated with a house to conduct village clinic*, *most of us are dwelling in rental houses*.*”* HSA, #RU06

The constrained resources heavily affect the performance of the IDSR system in the country.

*“At the beginning we are doing very well*. *WHO came and helped us to setup the system from 2002*, *we do supervision*, *training and so on*, *up until 2007 there is no fund*. *Government said we cannot take it*, *it’s too costly*.*”* ED, #MOH02

## Discussion

We assessed the differences between IDSR technical guideline and actual practice in the health system in Malawi for the first time. According to the quantitative data, we observed relatively good completeness of IDSR monthly reports and gradually improving of timeliness nationwide. Seasonality difference of timeliness was observed and can be related to the mode of reporting is mainly through physical paper transportation. The gaps and challenges affecting the surveillance quality were highlighted in the results and were similar to other African countries [12]. The actual case identification was replied on the volunteers of the VHC in Malawi. Hence, facility level IDSR reports may not be sufficiently timely to pick up the outbreaks from community. The strengthened community level surveillance and verbal autopsy to detect unusual deaths can be a good approach to detect lower level health events and provide timely response [30]. By the fact of traditional medicine health seeking behaviour among villagers in Malawi, it is also important to understand how these behaviours may delayed the outbreak detection.

Timeliness is a general problem to countries implementing IDSR system across Africa, and this makes the public health authorities unable to take quick action and respond to the suspected health events [12, 31]. African health ministries are fast adopting mHealth solutions to improve disease surveillance timeliness and capture real-time field data for surveillance and case management at the community level [32–34]. Tanzania piloted an IDSR reporting system using SMS function and regular phones for report in 2011 [35] and further expanded it to be the national strategy for diseases surveillance using Unstructured Supplementary Service Data (USSD) technology linked with DHIS2 for the immediate reporting for IDSR [33, 36, 37]. Zambia tried to use DHIS2 mobile to enhance its malaria surveillance in Lusaka district and to improve case management and reporting [38]. Other mobile technologies including smartphone applications, patient monitoring devices, Personal Digital Assistants (PDAs), as well as laptops and tablets PCs connected with network service were piloted and implemented in various African countries [39]. In Malawi, a pilot study conducted in Lilongwe District in Central Region showed that mobile technologies had good opportunities to improve timeliness of HMIS reports [40]. However, concerning the different purposes of HMIS and IDSR system, a more integrated electronic IDSR system is essential for the health authorities to correspond the diverse demands. Other notable issues were documented including technical, financial, infrastructural challenges, data security and medical supports during the design and implementation process of mHealth surveillance in sub-Saharan Africa countries [39]. Considering the complexity of public health works and needs of integration services at the community level [41], the utilization of mobile technologies requires more rigorous studies to evaluate such innovations for programme implementation to become sustainable and scalable [42].

Apart from mHealth solutions, researchers recommended to use syndromic surveillance approach combined with systematic virological testing as early as possible to maintain high quality situational awareness [43]. Several countries have established electronic data based syndromic surveillance systems to capture early warning signals of different diseases and health status especially related to respiratory infections [44–47]. However, electronic syndromic surveillance systems remain a novel technology for most of developing countries to adopt and implement [48]. Several EMR systems had been developed in Malawi and MOH decided to move towards a national standardized EMR system to support all levels of HMIS [49–51]. This provides a unique opportunity to utilize existing information technology and infrastructures to strengthen the IDSR system with nationwide syndromic surveillance. Yet it is critical to improve the user experiences of EMR users to improve the uptake and usage of the system. Similar countries can consider system synergies and existing infrastructure for IDSR enhancement.

We only focused on completeness and timeliness, and the accuracy attribute of the IDSR system performance was out of the scope of this study. However, according to the study result, we understand the inadequate laboratory infrastructure can affect the ability of case identification. Further clinical and laboratory data are needed for proper assessment. We only sampled one district to conduct qualitative assessment, however, we are confident that it is relevant for the Malawian context by the fact that the health care system is rather homogeneous in Malawi and the district we selected had a relatively good IDSR performance to generalize the implementation challenges.

## Conclusions

Lack of timeliness in reporting makes the IDSR system inoperative. Differences between IDSR technical guideline and actual practice existed in the current Malawian context. Shortcomings were due to financial constraints and poor basic infrastructure. However, the improving information technology infrastructure in Malawi, national standardized EMR system and emerging mHealth technologies can be opportunities for the country to overcome the challenges and improve the surveillance system.

## Supporting information

S1 Table Diseases, conditions or events requiring immediate reporting of Malawi IDSR system. (DOCX)

S2 Table Monthly IDSR reporting performance in Malawi in 2013. (DOCX)

S3 Table Monthly IDSR reporting completeness during the study period with seasonality and zone/district stratifications in Malawi. (DOCX)

S4 Table Monthly IDSR reporting timeliness during the study period with seasonality and zone/district stratifications in Malawi. (DOCX)

S5 Table The Malawi IDSR core functions and activities at each health system level comparing between guideline and practice. (DOCX)

S1 File Interview guide in English. (DOCX)

S2 File Interview guide in Chewa. (DOCX)

S3 File Interview guide in Tumbuka. (DOCX)

S4 File Qualitative data of interviews. (RAR)

S5 File Quantitative data of IDSR reporting rate summaries. (RAR)

## Abbreviations

AFRO: World Health Organization Regional Office for Africa
ARI: acute respiratory infections
CCM: community case management
CDC: Centers for Disease Control and Prevention
CHAM: Christian Health Association of Malawi
CMED: central monitoring and evaluation division
DEHO: district environmental health officer
DHIS: district health information system
DHIS2: district health information system 2
DHMT: district health management team
DHO: district health office
EMR: electronic medical records
EVD: Ebola virus disease
HCWs: health care workers
HMIS: health management information system
HSAs: health surveillance assistants
IDSR: integrated disease surveillance and response
IHR: international health regulation
mHealth: mobile health
MOH: Ministry of Health
PDAs: Personal Digital Assistants
SARS: severe acute respiratory syndrome
USSD: unstructured supplementary service data
VHC: village health committee
WHO: World Health Organization
