# Supplementary material for: Integrated Disease Surveillance and Response (IDSR) in Malawi: Implementation gaps and challenges for timely alert
Source: PLoS One. 2018 Nov 29;13(11):e0200858. doi: 10.1371/journal.pone.0200858 (PMC6264833; doi:10.1371/journal.pone.0200858)
Supplement: S1 Table — (DOCX) [file pone.0200858.s001.docx]

| **S1 Table: Diseases, conditions or events requiring immediate reporting of Malawi IDSR system [17]** | |
| --- | --- |
| - Acute Flaccid Paralysis (AFP) - Acute hemorrhagic fever syndrome (Ebola, Marburg, Lassa Fever, Rift Valley Fever (RVF), Crimean-Congo) - Adverse effects following immunization (AEFI) - Anthrax - Cholera - Cluster of SARI - Diarrhoea with blood (Shigella dysentery) - Influenza due to new subtype - Maternal death - Measles | - Meningococcal meningitis - Neonatal tetanus - Plague - Rabies (confirmed cases) - Severe Acute Respiratory Syndrome (SARS) - Smallpox - Typhoid fever - Yellow fever - Any public health event of international concern (infectious, zoonotic, food borne, chemical, radio nuclear or due to an unknown condition) |
