# Supplementary material for: Integrated Disease Surveillance and Response (IDSR) in Malawi: Implementation gaps and challenges for timely alert
Source: PLoS One. 2018 Nov 29;13(11):e0200858. doi: 10.1371/journal.pone.0200858 (PMC6264833; doi:10.1371/journal.pone.0200858)
Supplement: S2 Table — (DOCX) [file pone.0200858.s002.docx]

S2 Table: Monthly IDSR Reporting Performance in Malawi in 2013

| **Zone** | **District Name** | **Actual Reports** | **Expected Reports** | **Percent of Completed Reports (Completeness)** | **Reports on Time** | **Percent on Time (Timelines)** |
| --- | --- | --- | --- | --- | --- | --- |
| Central East Zone | Dowa | 39 | 264 | 14.8 | 0 | 0 |
|  | Kasungu | 6 | 84 | 7.1 | 0 | 0 |
|  | Nkhotakota | 58 | 252 | 23 | 0 | 0 |
|  | Ntchisi | 30 | 156 | 19.2 | 0 | 0 |
|  | Salima | 102 | 228 | 44.7 | 0 | 0 |
|  | **Total** | **235** | **984** | **23.9** | **0** | **0** |
| Central West Zone | Dedza | 374 | 408 | 91.7 | 26 | 6.4 |
|  | Lilongwe | 418 | 624 | 67 | 94 | 15.1 |
|  | Mchinji | 33 | 216 | 15.3 | 5 | 2.3 |
|  | Ntcheu | 74 | 468 | 15.8 | 0 | 0 |
|  | **Total** | **899** | **1,716** | **52.4** | **125** | **7.3** |
| North Zone | Chitipa | 107 | 144 | 74.3 | 20 | 13.9 |
|  | Karonga | 147 | 228 | 64.5 | 9 | 3.9 |
|  | Likoma | 0 | 24 | 0 | 0 | 0 |
|  | Mzimba-North | 253 | 300 | 84.3 | 99 | 33 |
|  | Mzimba-South | 54 | 384 | 14.1 | 21 | 5.5 |
|  | Rumphi* | 204 | 204 | 100 | 57 | 27.9 |
|  | **Total** | **765** | **1,284** | **59.6** | **206** | **16** |
| South East Zone | Balaka | 124 | 192 | 64.6 | 21 | 10.9 |
|  | Machinga | 8 | 252 | 3.2 | 0 | 0 |
|  | Mangochi | 58 | 504 | 11.5 | 18 | 3.6 |
|  | Mulanje | 77 | 276 | 27.9 | 4 | 1.4 |
|  | Phalombe | 1 | 12 | 8.3 | 0 | 0 |
|  | Zomba | 9 | 432 | 2.1 | 0 | 0 |
|  | **Total** | **277** | **1,668** | **16.6** | **43** | **2.6** |
| South West Zone | Blantyre | 0 | 384 | 0 | 0 | 0 |
|  | Chikwawa | 3 | 348 | 0.9 | 0 | 0 |
|  | Mwanza | 0 | 48 | 0 | 0 | 0 |
|  | Neno | 53 | 180 | 29.4 | 8 | 4.4 |
|  | Nsanje | 5 | 264 | 1.9 | 2 | 0.8 |
|  | Thyolo | 107 | 384 | 27.9 | 0 | 0 |
|  | **Total** | **168** | **1,608** | **10.4** | **10** | **0.6** |
| **National Total** | | **2,344** | **7,452** | **31.5** | **384** | **5.2** |

*The district was chosen for study by the fact of best performance among all districts in Malawi in 2013
