# Supplementary material for: Integrated Disease Surveillance and Response (IDSR) in Malawi: Implementation gaps and challenges for timely alert
Source: PLoS One. 2018 Nov 29;13(11):e0200858. doi: 10.1371/journal.pone.0200858 (PMC6264833; doi:10.1371/journal.pone.0200858)
Supplement: S3 Table — (DOCX) [file pone.0200858.s003.docx]

**S3 Table - Monthly IDSR reporting completeness performance during the study period with seasonality and zone/district stratifications in Malawi**

| Year/Period | 2014 | | | | | | | | 2015 | | | | | | | |
| --- | --- | --- | --- | --- | --- | --- | --- | --- | --- | --- | --- | --- | --- | --- | --- | --- |
| Seasonality | Dry Season | | Rainy Season | | Total | | Z-Score 1 | Z-Score 2 | Dry Season | | Rainy Season | | Total | | Z-Score 1 | Z-Score 2 |
| Name of Zone/District | # of Expected Reports | Completeness  (%) | # of Expected Reports | Completeness  (%) | # of Expected Reports | Completeness  (%) |  |  | # of Expected Reports | Completeness  (%) | # of Expected Reports | Completeness  (%) | # of Expected Reports | Completeness  (%) |  |  |
| Central East Zone | 82 | 65.9% | 164 | 48.8% | 246 | 54.5% | 2.535^†^ | -4.570^‡^ | 492 | 63.6% | 492 | 59.3% | 984 | 61.5% | 1.376 | -8.358^‡^ |
| Dowa | 22 | 50.0% | 44 | 25.0% | 66 | 33.3% | 2.031^†^ | -6.143^‡^ | 132 | 50.8% | 132 | 47.7% | 264 | 49.2% | 0.492 | -8.192^‡^ |
| Kasungu | 7 | 71.4% | 14 | 64.3% | 21 | 66.7% | 0.327 | -0.225 | 42 | 97.6% | 42 | 92.9% | 84 | 95.2% | 1.025 | 8.947^‡^ |
| Nkhotakota | 21 | 66.7% | 42 | 50.0% | 63 | 55.6% | 1.255 | -2.145^†^ | 126 | 63.5% | 126 | 52.4% | 252 | 57.9% | 1.787 | -5.310^‡^ |
| Ntchisi | 13 | 38.5% | 26 | 23.1% | 39 | 28.2% | 1.007 | -5.659^‡^ | 78 | 33.3% | 78 | 26.9% | 156 | 30.1% | 0.873 | -12.065^‡^ |
| Salima | 19 | 100.0% | 38 | 86.8% | 57 | 91.2% | 1.655 | 5.938^‡^ | 114 | 86.8% | 114 | 90.4% | 228 | 88.6% | -0.833 | 6.721^‡^ |
| Central West Zone | 143 | 86.0% | 286 | 85.3% | 429 | 85.5% | 0.194 | 9.759^‡^ | 858 | 78.8% | 858 | 82.9% | 1,716 | 80.8% | -2.146^†^ | 6.712^‡^ |
| Dedza | 34 | 97.1% | 68 | 97.1% | 102 | 97.1% | 0 | 16.784^‡^ | 204 | 83.3% | 204 | 89.2% | 408 | 86.3% | -1.726 | 6.941^‡^ |
| Lilongwe | 52 | 84.6% | 104 | 84.6% | 156 | 84.6% | 0 | 5.412^‡^ | 312 | 82.7% | 312 | 88.1% | 624 | 85.4% | -1.928 | 7.763^‡^ |
| Mchinji | 18 | 44.4% | 36 | 38.9% | 54 | 40.7% | 0.392 | -4.224^‡^ | 108 | 28.7% | 108 | 28.7% | 216 | 28.7% | 0 | -14.862^‡^ |
| Ntcheu | 39 | 97.4% | 78 | 97.4% | 117 | 97.4% | 0.000 | 19.473^‡^ | 234 | 92.7% | 234 | 95.3% | 468 | 94.0% | -1.169 | 17.849^‡^ |
| North Zone | 107 | 87.9% | 214 | 91.1% | 321 | 90.0% | -0.922 | 12.589^‡^ | 642 | 88.8% | 642 | 88.9% | 1,284 | 88.9% | -0.089 | 16.418^‡^ |
| Chitipa | 12 | 75.0% | 24 | 75.0% | 36 | 75.0% | 0 | 0.834 | 72 | 72.2% | 72 | 70.8% | 144 | 71.5% | 0.185 | -0.777 |
| Karonga | 19 | 89.5% | 38 | 94.7% | 57 | 93.0% | -0.733 | 7.094^‡^ | 114 | 78.1% | 114 | 82.5% | 228 | 80.3% | -0.832 | 2.206^†^ |
| Likoma | 2 | 100.0% | 4 | 100.0% | 6 | 100.0% | N/A | N/A | 12 | 100.0% | 12 | 100.0% | 24 | 100.0% | N/A | N/A |
| Mzimba-North | 25 | 76.0% | 50 | 82.0% | 75 | 80.0% | -0.612 | 2.386^†^ | 150 | 96.0% | 150 | 99.3% | 300 | 97.7% | -1.912 | 26.639^‡^ |
| Mzimba-South | 32 | 96.9% | 64 | 96.9% | 96 | 96.9% | 0.000 | 15.708^‡^ | 192 | 90.6% | 192 | 93.2% | 384 | 91.9% | -0.937 | 12.573^‡^ |
| Rumphi^*^ | 17 | 94.1% | 34 | 100.0% | 51 | 98.0% | -1.428 | 14.967^‡^ | 102 | 97.1% | 102 | 84.3% | 204 | 90.7% | 3.132^†^ | 7.980^‡^ |
| South East Zone | 139 | 69.8% | 278 | 69.1% | 417 | 69.3% | 0.150 | 0.143 | 834 | 73.6% | 834 | 72.3% | 1,668 | 73.0% | 0.606 | -1.368 |
| Balaka | 16 | 87.5% | 32 | 87.5% | 48 | 87.5% | 0 | 3.880^‡^ | 96 | 76.0% | 96 | 83.3% | 192 | 79.7% | -1.256 | 1.804^†^ |
| Machinga | 21 | 100.0% | 42 | 100.0% | 63 | 100.0% | N/A | N/A | 126 | 98.4% | 126 | 96.8% | 252 | 97.6% | 0.826 | 24.126^‡^ |
| Mangochi | 42 | 76.2% | 84 | 73.8% | 126 | 74.6% | 0.289 | 1.450 | 252 | 70.2% | 252 | 69.0% | 504 | 69.6% | 0.290 | -2.347^†^ |
| Mulanje | 23 | 65.2% | 46 | 73.9% | 69 | 71.0% | -0.751 | 0.372 | 138 | 76.1% | 138 | 76.8% | 276 | 76.4% | -0.142 | 0.783 |
| Phalombe | 1 | 0.0% | 2 | 0.0% | 3 | 0.0% | N/A | N/A | 6 | 83.3% | 6 | 66.7% | 12 | 75.0% | 0.667 | 0.044 |
| Zomba | 36 | 41.7% | 72 | 36.1% | 108 | 38.0% | 0.561 | -6.642^‡^ | 216 | 60.2% | 216 | 54.2% | 432 | 57.2% | 1.264 | -7.255^‡^ |
| South West Zone | 134 | 45.5% | 268 | 41.8% | 402 | 43.0% | 0.712 | -10.507^‡^ | 804 | 69.8% | 804 | 61.4% | 1,608 | 65.6% | 3.518^†^ | -7.462^‡^ |
| Blantyre | 32 | 0.0% | 64 | 0.0% | 96 | 0.0% | N/A | N/A | 192 | 89.6% | 192 | 54.7% | 384 | 72.1% | 7.626^‡^ | -1.011 |
| Chikwawa | 29 | 62.1% | 58 | 58.6% | 87 | 59.8% | 0.309 | -1.752^†^ | 174 | 56.3% | 174 | 52.9% | 348 | 54.6% | 0.646 | -7.438^‡^ |
| Mwanza | 4 | 100.0% | 8 | 100.0% | 12 | 100.0% | N/A | N/A | 24 | 100.0% | 24 | 95.8% | 48 | 97.9% | 1.011 | 11.384^‡^ |
| Neno | 15 | 46.7% | 30 | 36.7% | 45 | 40.0% | 0.646 | -3.968^‡^ | 90 | 60.0% | 90 | 53.3% | 180 | 56.7% | 0.903 | -4.815^‡^ |
| Nsanje | 22 | 72.7% | 44 | 72.7% | 66 | 72.7% | 0 | 0.683 | 132 | 95.5% | 132 | 89.4% | 264 | 92.4% | 1.861 | 11.038^‡^ |
| Thyolo | 32 | 50.0% | 64 | 42.2% | 96 | 44.8% | 0.726 | -4.766^‡^ | 192 | 45.3% | 192 | 56.3% | 384 | 50.8% | -2.144^†^ | -9.277^‡^ |
| National Total | 605 | 70.9% | 1,210 | 68.0% | 1,815 | 69.0% | 1.256 |  | 3,630 | 75.3% | 3,630 | 73.6% | 7,260 | 74.4% | 1.695 |  |

| Year/Period | 2016 | | | | | | | | Total studied period (Oct.2014-Sep.2016) | | | | | | | |
| --- | --- | --- | --- | --- | --- | --- | --- | --- | --- | --- | --- | --- | --- | --- | --- | --- |
| Seasonality | Dry Season | | Rainy Season | | Total | | Z-Score 1 | Z-Score 2 | Dry Season | | Rainy Season | | Total | | Z-Score 1 | Z-Score 2 |
| Name of Zone/District | # of Expected Reports | Completeness  (%) | # of Expected Reports | Completeness  (%) | # of Expected Reports | Completeness  (%) |  |  | # of Expected Reports | Completeness  (%) | # of Expected Reports | Completeness  (%) | # of Expected Reports | Completeness  (%) |  |  |
| Central East Zone | 410 | 62.4% | 328 | 60.4% | 738 | 61.5% | 0.575 | -6.259^‡^ | 984 | 63.3% | 984 | 57.9% | 1,968 | 60.6% | 2.445^†^ | -11.349^‡^ |
| Dowa | 110 | 48.2% | 88 | 55.7% | 198 | 51.5% | -1.049 | -5.972^‡^ | 264 | 49.6% | 264 | 46.6% | 528 | 48.1% | 0.697 | -11.504^‡^ |
| Kasungu | 35 | 80.0% | 28 | 89.3% | 63 | 84.1% | -1.002 | 2.476^†^ | 84 | 88.1% | 84 | 86.9% | 168 | 87.5% | 0.233 | 5.636^‡^ |
| Nkhotakota | 105 | 60.0% | 84 | 64.3% | 189 | 61.9% | -0.603 | -3.064^†^ | 252 | 62.3% | 252 | 56.0% | 504 | 59.1% | 1.450 | -6.390^‡^ |
| Ntchisi | 65 | 66.2% | 52 | 21.2% | 117 | 46.2% | 4.852^‡^ | -5.766^‡^ | 156 | 47.4% | 156 | 24.4% | 312 | 35.9% | 4.249^‡^ | -13.706^‡^ |
| Salima | 95 | 72.6% | 76 | 77.6% | 171 | 74.9% | -0.749 | 0.641 | 228 | 82.0% | 228 | 85.5% | 456 | 83.8% | -1.016 | 6.169^‡^ |
| Central West Zone | 715 | 68.8% | 572 | 71.7% | 1,287 | 70.1% | -1.116 | -2.070^†^ | 1,716 | 75.2% | 1,716 | 79.5% | 3,432 | 77.4% | -3.020^†^ | 5.979^‡^ |
| Dedza | 170 | 70.0% | 136 | 70.6% | 306 | 70.3% | -0.112 | -0.944 | 408 | 78.9% | 408 | 84.3% | 816 | 81.6% | -1.988^†^ | 6.267^‡^ |
| Lilongwe | 260 | 67.7% | 208 | 71.2% | 468 | 69.2% | -0.806 | -1.639 | 624 | 76.6% | 624 | 81.9% | 1,248 | 79.2% | -2.303^†^ | 5.337^‡^ |
| Mchinji | 90 | 45.6% | 72 | 41.7% | 162 | 43.8% | 0.496 | -7.414^‡^ | 216 | 37.0% | 216 | 34.7% | 432 | 35.9% | 0.502 | -16.137^‡^ |
| Ntcheu | 195 | 80.0% | 156 | 87.2% | 351 | 83.2% | -1.787 | 5.242^‡^ | 468 | 87.8% | 468 | 92.9% | 936 | 90.4% | -2.661^†^ | 17.917^‡^ |
| North Zone | 535 | 86.2% | 428 | 89.5% | 963 | 87.6% | -1.555 | 14.065^‡^ | 1,284 | 87.6% | 1,284 | 89.5% | 2,568 | 88.6% | -1.487 | 24.560^‡^ |
| Chitipa | 60 | 80.0% | 48 | 81.3% | 108 | 80.6% | -0.163 | 2.056^†^ | 144 | 75.7% | 144 | 75.0% | 288 | 75.3% | 0.137 | 0.877 |
| Karonga | 95 | 83.2% | 76 | 81.6% | 171 | 82.5% | 0.270 | 3.345^†^ | 228 | 81.1% | 228 | 84.2% | 456 | 82.7% | -0.866 | 5.392^‡^ |
| Likoma | 10 | 100.0% | 8 | 100.0% | 18 | 100.0% | N/A | N/A | 24 | 100.0% | 24 | 100.0% | 48 | 100.0% | N/A | N/A |
| Mzimba-North | 125 | 94.4% | 100 | 97.0% | 225 | 95.6% | -0.940 | 16.616^‡^ | 300 | 93.7% | 300 | 95.7% | 600 | 94.7% | -1.090 | 23.489^‡^ |
| Mzimba-South | 160 | 76.9% | 128 | 85.9% | 288 | 80.9% | -1.944 | 3.530^†^ | 384 | 85.4% | 384 | 91.4% | 768 | 88.4% | -2.593^†^ | 13.239^‡^ |
| Rumphi^*^ | 85 | 97.6% | 68 | 98.5% | 153 | 98.0% | -0.391 | 22.582^‡^ | 204 | 97.1% | 204 | 91.7% | 408 | 94.4% | 2.361^†^ | 18.604^‡^ |
| South East Zone | 695 | 69.2% | 556 | 74.6% | 1,251 | 71.6% | -2.118^†^ | -0.867 | 1,668 | 71.5% | 1,668 | 72.5% | 3,336 | 72.0% | -0.694 | -1.438 |
| Balaka | 80 | 73.8% | 64 | 76.6% | 144 | 75.0% | -0.387 | 0.630 | 192 | 76.0% | 192 | 81.8% | 384 | 78.9% | -1.376 | 2.779 |
| Machinga | 105 | 78.1% | 84 | 95.2% | 189 | 85.7% | -3.347^†^ | 5.102^‡^ | 252 | 90.1% | 252 | 96.8% | 504 | 93.5% | -3.061^†^ | 18.453^‡^ |
| Mangochi | 210 | 55.2% | 168 | 61.3% | 378 | 57.9% | -1.188 | -5.825^‡^ | 504 | 64.5% | 504 | 67.3% | 1,008 | 65.9% | -0.930 | -4.853^‡^ |
| Mulanje | 115 | 89.6% | 92 | 94.6% | 207 | 91.8% | -1.302 | 9.988^‡^ | 276 | 80.8% | 276 | 82.2% | 552 | 81.5% | -0.439 | 5.086^‡^ |
| Phalombe | 5 | 60.0% | 4 | 0.0% | 9 | 33.3% | 1.897 | -2.507^†^ | 12 | 66.7% | 12 | 33.3% | 24 | 50.0% | 1.633 | -2.265^†^ |
| Zomba | 180 | 65.6% | 144 | 66.7% | 324 | 66.0% | -0.210 | -2.538^†^ | 432 | 60.9% | 432 | 55.3% | 864 | 58.1% | 1.655 | -8.947^‡^ |
| South West Zone | 670 | 73.1% | 536 | 69.8% | 1,206 | 71.6% | 1.286 | -0.836 | 1,608 | 69.2% | 1,608 | 60.9% | 3,216 | 65.0% | 4.882^‡^ | -9.598^‡^ |
| Blantyre | 160 | 81.3% | 128 | 85.9% | 288 | 83.3% | -1.061 | 4.830^‡^ | 384 | 78.6% | 384 | 56.0% | 768 | 67.3% | 6.693^‡^ | -3.428^†^ |
| Chikwawa | 145 | 52.4% | 116 | 54.3% | 261 | 53.3% | -0.305 | -6.305^‡^ | 348 | 55.2% | 348 | 54.3% | 696 | 54.7% | 0.229 | -9.741^‡^ |
| Mwanza | 20 | 95.0% | 16 | 100.0% | 36 | 97.2% | -0.907 | 8.943^‡^ | 48 | 97.9% | 48 | 97.9% | 96 | 97.9% | 0 | 17.011^‡^ |
| Neno | 75 | 52.0% | 60 | 60.0% | 135 | 55.6% | -0.930 | -4.015^‡^ | 180 | 55.6% | 180 | 52.8% | 360 | 54.2% | 0.529 | -7.217^‡^ |
| Nsanje | 110 | 80.9% | 88 | 94.3% | 198 | 86.9% | -2.776^†^ | 5.892^‡^ | 264 | 87.5% | 264 | 88.3% | 528 | 87.9% | -0.267 | 10.391^‡^ |
| Thyolo | 160 | 85.6% | 128 | 51.6% | 288 | 70.5% | 6.298^‡^ | -0.8340 | 384 | 62.5% | 384 | 52.3% | 768 | 57.4% | 2.846^†^ | -8.798^‡^ |
| National Total | 3,025 | 72.1% | 2,420 | 73.6% | 5,445 | 72.7% | -1.225 |  | 7,260 | 73.6% | 7,260 | 72.6% | 14,520 | 73.1% | 1.292 |  |

Z-Score 1: Difference of report completeness between dry season and rainy season within the national, zonal and district strata with two-tailed hypothesis (α=0.05, Z ≥ 1.960 or Z ≤ -1.960)

Z-Score 2: Difference of report completeness between zone/district annual performance and the national average with one-tailed hypothesis (α=0.05, Z ≥ 1.645 or Z ≤ -1.645)

^*^Studied district  ^†^ P-value < 0.05 ^‡^ P-value < 0.0001
