# Supplementary material for: Integrated Disease Surveillance and Response (IDSR) in Malawi: Implementation gaps and challenges for timely alert
Source: PLoS One. 2018 Nov 29;13(11):e0200858. doi: 10.1371/journal.pone.0200858 (PMC6264833; doi:10.1371/journal.pone.0200858)
Supplement: S4 Table — (DOCX) [file pone.0200858.s004.docx]

**S4 Table - Monthly IDSR reporting timeliness performance during the study period with seasonality and zone/district stratifications in Malawi**

| Year/Period | 2014 | | | | | | | | 2015 | | | | | | | |
| --- | --- | --- | --- | --- | --- | --- | --- | --- | --- | --- | --- | --- | --- | --- | --- | --- |
| Seasonality | Dry Season | | Rainy Season | | Total | | Z-Score 1 | Z-Score 2 | Dry Season | | Rainy Season | | Total | | Z-Score 1 | Z-Score 2 |
| Name of Zone/District | # of Expected Reports | Timeliness  (%) | # of Expected Reports | Timeliness  (%) | # of Expected Reports | Timeliness  (%) |  |  | # of Expected Reports | Timeliness  (%) | # of Expected Reports | Timeliness  (%) | # of Expected Reports | Timeliness  (%) |  |  |
| Central East Zone | 82 | 31.7% | 164 | 14.6% | 246 | 20.3% | 3.137^†^ | -5.736^‡^ | 492 | 27.8% | 492 | 21.3% | 984 | 24.6% | 2.369^†^ | -9.436^‡^ |
| Dowa | 22 | 27.3% | 44 | 9.1% | 66 | 15.2% | 1.942 | -4.507^‡^ | 132 | 18.2% | 132 | 22.7% | 264 | 20.5% | -0.915 | -6.885^‡^ |
| Kasungu | 7 | 0.0% | 14 | 14.3% | 21 | 9.5% | -1.051 | -3.984^‡^ | 42 | 42.9% | 42 | 9.5% | 84 | 26.2% | 3.474^†^ | -2.368^†^ |
| Nkhotakota | 21 | 47.6% | 42 | 23.8% | 63 | 31.7% | 1.914 | -0.562 | 126 | 19.8% | 126 | 25.4% | 252 | 22.6% | -1.054 | -5.665^‡^ |
| Ntchisi | 13 | 30.8% | 26 | 7.7% | 39 | 15.4% | 1.883 | -3.402^†^ | 78 | 21.8% | 78 | 11.5% | 156 | 16.7% | 1.719 | -6.998^‡^ |
| Salima | 19 | 31.6% | 38 | 15.8% | 57 | 21.1% | 1.378 | -2.591^†^ | 114 | 46.5% | 114 | 26.3% | 228 | 36.4% | 3.166^†^ | -0.359 |
| Central West Zone | 143 | 73.4% | 286 | 59.4% | 429 | 64.1% | 2.847^†^ | 12.548^‡^ | 858 | 60.4% | 858 | 47.6% | 1,716 | 54.0% | 5.328^‡^ | 13.642^‡^ |
| Dedza | 34 | 85.3% | 68 | 95.6% | 102 | 92.2% | -1.823 | 21.456^‡^ | 204 | 77.0% | 204 | 69.6% | 408 | 73.3% | 1.678 | 16.314^‡^ |
| Lilongwe | 52 | 61.5% | 104 | 39.4% | 156 | 46.8% | 2.610^†^ | 2.942^†^ | 312 | 44.2% | 312 | 26.0% | 624 | 35.1% | 4.781^‡^ | -1.283 |
| Mchinji | 18 | 33.3% | 36 | 22.2% | 54 | 25.9% | 0.878 | -1.529 | 108 | 16.7% | 108 | 4.6% | 216 | 10.6% | 2.868^†^ | -12.817^‡^ |
| Ntcheu | 39 | 97.4% | 78 | 71.8% | 117 | 80.3% | 3.290^†^ | 12.330^‡^ | 234 | 87.6% | 234 | 76.9% | 468 | 82.3% | 3.025^†^ | 25.326^‡^ |
| North Zone | 107 | 15.9% | 214 | 38.3% | 321 | 30.8% | -4.102^‡^ | -1.629 | 642 | 33.0% | 642 | 31.3% | 1,284 | 32.2% | 0.657 | -4.129^‡^ |
| Chitipa | 12 | 58.3% | 24 | 33.3% | 36 | 41.7% | 1.434 | 0.806 | 72 | 30.6% | 72 | 23.6% | 144 | 27.1% | 0.938 | -2.826^†^ |
| Karonga | 19 | 36.8% | 38 | 42.1% | 57 | 40.4% | -0.382 | 0.817 | 114 | 15.8% | 114 | 36.8% | 228 | 26.3% | -3.610^†^ | -3.852^‡^ |
| Likoma | 2 | 0.0% | 4 | 50.0% | 6 | 33.3% | -1.225 | -0.089 | 12 | 50.0% | 12 | 41.7% | 24 | 45.8% | 0.410 | 0.815 |
| Mzimba-North | 25 | 8.0% | 50 | 76.0% | 75 | 53.3% | -5.565^‡^ | 3.175^†^ | 150 | 64.7% | 150 | 65.3% | 300 | 65.0% | -0.121 | 9.969^‡^ |
| Mzimba-South | 32 | 0.0% | 64 | 14.1% | 96 | 9.4% | -2.228^†^ | -8.628^‡^ | 192 | 15.1% | 192 | 18.8% | 384 | 16.9% | -0.953 | -10.776^‡^ |
| Rumphi^*^ | 17 | 5.9% | 34 | 26.5% | 51 | 19.6% | -1.746 | -2.776^†^ | 102 | 39.2% | 102 | 2.9% | 204 | 21.1% | 6.351^‡^ | -5.767^‡^ |
| South East Zone | 139 | 35.3% | 278 | 41.0% | 417 | 39.1% | -1.135 | 1.694^†^ | 834 | 50.4% | 834 | 35.0% | 1,668 | 42.7% | 6.336^‡^ | 4.242^‡^ |
| Balaka | 16 | 62.5% | 32 | 87.5% | 48 | 79.2% | -2.010^†^ | 7.528^‡^ | 96 | 66.7% | 96 | 64.6% | 192 | 65.6% | 0.304 | 8.191^‡^ |
| Machinga | 21 | 61.9% | 42 | 83.3% | 63 | 76.2% | -1.882 | 7.668^‡^ | 126 | 81.0% | 126 | 65.9% | 252 | 73.4% | 2.709^†^ | 12.887^‡^ |
| Mangochi | 42 | 45.2% | 84 | 38.1% | 126 | 40.5% | 0.770 | 1.243 | 252 | 48.8% | 252 | 32.1% | 504 | 40.5% | 3.811^†^ | 1.339 |
| Mulanje | 23 | 0.0% | 46 | 13.0% | 69 | 8.7% | -1.813 | -7.767^‡^ | 138 | 65.2% | 138 | 30.4% | 276 | 47.8% | 5.784^‡^ | 3.418^†^ |
| Phalombe | 1 | 0.0% | 2 | 0.0% | 3 | 0.0% | N/A | N/A | 6 | 33.3% | 6 | 50.0% | 12 | 41.7% | -0.586 | 0.289 |
| Zomba | 36 | 19.4% | 72 | 18.1% | 108 | 18.5% | 0.175 | -4.420^‡^ | 216 | 18.1% | 216 | 9.7% | 432 | 13.9% | 2.504^†^ | -14.219^‡^ |
| South West Zone | 134 | 17.2% | 268 | 9.7% | 402 | 12.2% | 2.156^†^ | -14.005^‡^ | 804 | 29.7% | 804 | 24.1% | 1,608 | 26.9% | 2.530^†^ | -9.601^‡^ |
| Blantyre | 32 | 0.0% | 64 | 0.0% | 96 | 0.0% | N/A | N/A | 192 | 57.8% | 192 | 39.1% | 384 | 48.4% | 3.676^†^ | 4.270^‡^ |
| Chikwawa | 29 | 13.8% | 58 | 8.6% | 87 | 10.3% | 0.747 | -7.564^‡^ | 174 | 14.4% | 174 | 12.6% | 348 | 13.5% | 0.471 | -13.122^‡^ |
| Mwanza | 4 | 0.0% | 8 | 50.0% | 12 | 33.3% | -1.732 | -0.126 | 24 | 75.0% | 24 | 37.5% | 48 | 56.3% | 2.619^†^ | 2.612^†^ |
| Neno | 15 | 40.0% | 30 | 30.0% | 45 | 33.3% | 0.671 | -0.243 | 90 | 42.2% | 90 | 35.6% | 180 | 38.9% | 0.917 | 0.369 |
| Nsanje | 22 | 0.0% | 44 | 18.2% | 66 | 12.1% | -2.133^†^ | -5.705^‡^ | 132 | 29.5% | 132 | 25.0% | 264 | 27.3% | 0.829 | -3.749^‡^ |
| Thyolo | 32 | 40.6% | 64 | 0.0% | 96 | 13.5% | 5.484^‡^ | -6.156^‡^ | 192 | 4.2% | 192 | 12.0% | 384 | 8.1% | -2.810^†^ | -21.202^‡^ |
| National Total | 605 | 36.4% | 1,210 | 34.4% | 1,815 | 35.0% | 0.835 |  | 3,630 | 42.0% | 3,630 | 33.1% | 7,260 | 37.5% | 7.901^‡^ |  |

| Year/Period | 2016 | | | | | | | | Total studied period (Oct.2014-Sep.2016) | | | | | | | |
| --- | --- | --- | --- | --- | --- | --- | --- | --- | --- | --- | --- | --- | --- | --- | --- | --- |
| Seasonality | Dry Season | | Rainy Season | | Total | | Z-Score 1 | Z-Score 2 | Dry Season | | Rainy Season | | Total | | Z-Score 1 | Z-Score 2 |
| Name of Zone/District | # of Expected Reports | Timeliness  (%) | # of Expected Reports | Timeliness  (%) | # of Expected Reports | Timeliness  (%) |  |  | # of Expected Reports | Timeliness  (%) | # of Expected Reports | Timeliness  (%) | # of Expected Reports | Timeliness  (%) |  |  |
| Central East Zone | 410 | 32.0% | 328 | 27.4% | 738 | 29.9% | 1.330 | -9.199^‡^ | 984 | 29.9% | 984 | 22.3% | 1,968 | 26.1% | 3.851^‡^ | -14.281^‡^ |
| Dowa | 110 | 28.2% | 88 | 29.5% | 198 | 28.8% | -0.211 | -5.180^‡^ | 264 | 23.1% | 264 | 22.7% | 528 | 22.9% | 0.104 | -9.449^‡^ |
| Kasungu | 35 | 14.3% | 28 | 0.0% | 63 | 7.9% | 2.084^†^ | -11.017^‡^ | 84 | 27.4% | 84 | 7.1% | 168 | 17.3% | 3.471^†^ | -7.867^‡^ |
| Nkhotakota | 105 | 35.2% | 84 | 31.0% | 189 | 33.3% | 0.621 | -3.535^†^ | 252 | 28.6% | 252 | 27.0% | 504 | 27.8% | 0.398 | -6.226^‡^ |
| Ntchisi | 65 | 61.5% | 52 | 17.3% | 117 | 41.9% | 4.819^‡^ | -0.784 | 156 | 39.1% | 156 | 12.8% | 312 | 26.0% | 5.294^‡^ | -5.736^‡^ |
| Salima | 95 | 18.9% | 76 | 38.2% | 171 | 27.5% | -2.796^†^ | -5.263^‡^ | 228 | 33.8% | 228 | 28.5% | 456 | 31.1% | 1.214 | -4.178^‡^ |
| Central West Zone | 715 | 55.2% | 572 | 55.6% | 1,287 | 55.4% | -0.125 | 7.178^‡^ | 1,716 | 59.3% | 1,716 | 52.2% | 3,432 | 55.8% | 4.193^‡^ | 18.365^‡^ |
| Dedza | 170 | 64.1% | 136 | 51.5% | 306 | 58.5% | 2.231^†^ | 4.630^‡^ | 408 | 72.3% | 408 | 67.9% | 816 | 70.1% | 1.376 | 18.655^‡^ |
| Lilongwe | 260 | 43.5% | 208 | 51.4% | 468 | 47.0% | -1.719 | 0.674 | 624 | 45.4% | 624 | 36.7% | 1,248 | 41.0% | 3.108^†^ | 0.593 |
| Mchinji | 90 | 28.9% | 72 | 18.1% | 162 | 24.1% | 1.603 | -6.365^‡^ | 216 | 23.1% | 216 | 12.0% | 432 | 17.6% | 3.033^†^ | -12.341^‡^ |
| Ntcheu | 195 | 75.4% | 156 | 82.1% | 351 | 78.3% | -1.507 | 14.962^‡^ | 468 | 83.3% | 468 | 77.8% | 936 | 80.6% | 2.147^†^ | 31.196^‡^ |
| North Zone | 535 | 50.1% | 428 | 48.6% | 963 | 49.4% | 0.461 | 2.467^†^ | 1,284 | 38.7% | 1,284 | 38.2% | 2,568 | 38.5% | 0.243 | -1.798^†^ |
| Chitipa | 60 | 20.0% | 48 | 41.7% | 108 | 29.6% | -2.450^†^ | -3.602^†^ | 144 | 28.5% | 144 | 31.3% | 288 | 29.9% | -0.515 | -3.834^‡^ |
| Karonga | 95 | 53.7% | 76 | 30.3% | 171 | 43.3% | 3.072^†^ | -0.575 | 228 | 33.3% | 228 | 35.5% | 456 | 34.4% | -0.493 | -2.593^†^ |
| Likoma | 10 | 90.0% | 8 | 87.5% | 18 | 88.9% | 0.168 | 5.864^‡^ | 24 | 62.5% | 24 | 58.3% | 48 | 60.4% | 0.295 | 2.864^†^ |
| Mzimba-North | 125 | 48.0% | 100 | 52.0% | 225 | 49.8% | -0.596 | 1.297 | 300 | 53.0% | 300 | 62.7% | 600 | 57.8% | -2.397^†^ | 8.747^‡^ |
| Mzimba-South | 160 | 53.1% | 128 | 52.3% | 288 | 52.8% | 0.132 | 2.489^†^ | 384 | 29.7% | 384 | 29.2% | 768 | 29.4% | 0.158 | -6.551^‡^ |
| Rumphi^*^ | 85 | 60.0% | 68 | 57.4% | 153 | 58.8% | 0.331 | 3.360^†^ | 204 | 45.1% | 204 | 25.0% | 408 | 35.0% | 4.254^‡^ | -2.181^†^ |
| South East Zone | 695 | 44.6% | 556 | 49.5% | 1,251 | 46.8% | -1.711 | 0.927 | 1,668 | 46.7% | 1,668 | 40.8% | 3,336 | 43.8% | 3.420^†^ | 4.151^‡^ |
| Balaka | 80 | 66.3% | 64 | 73.4% | 144 | 69.4% | -0.930 | 6.250^‡^ | 192 | 66.1% | 192 | 71.4% | 384 | 68.8% | -1.101 | 12.070^‡^ |
| Machinga | 105 | 51.4% | 84 | 59.5% | 189 | 55.0% | -1.112 | 2.645^†^ | 252 | 67.1% | 252 | 66.7% | 504 | 66.9% | 0.095 | 12.718^‡^ |
| Mangochi | 210 | 46.2% | 168 | 41.1% | 378 | 43.9% | 0.997 | -0.603 | 504 | 47.4% | 504 | 36.1% | 1,008 | 41.8% | 3.640^†^ | 1.008 |
| Mulanje | 115 | 52.2% | 92 | 66.3% | 207 | 58.5% | -2.050^†^ | 3.795^‡^ | 276 | 54.3% | 276 | 39.5% | 552 | 46.9% | 3.497^†^ | 3.164^†^ |
| Phalombe | 5 | 0.0% | 4 | 0.0% | 9 | 0.0% | N/A | N/A | 12 | 16.7% | 12 | 25.0% | 24 | 20.8% | -0.503 | -2.336^†^ |
| Zomba | 180 | 25.6% | 144 | 33.3% | 324 | 29.0% | -1.533 | -6.522^‡^ | 432 | 21.3% | 432 | 19.0% | 864 | 20.1% | 0.848 | -14.703^‡^ |
| South West Zone | 670 | 44.0% | 536 | 34.5% | 1,206 | 39.8% | 3.354^†^ | -4.011^‡^ | 1,608 | 34.6% | 1,608 | 25.2% | 3,216 | 29.9% | 5.854^‡^ | -12.741^‡^ |
| Blantyre | 160 | 50.0% | 128 | 60.9% | 288 | 54.9% | -1.853 | 3.208^†^ | 384 | 49.7% | 384 | 39.8% | 768 | 44.8% | 2.757^†^ | 2.559^†^ |
| Chikwawa | 145 | 26.2% | 116 | 22.4% | 261 | 24.5% | 0.708 | -7.861^‡^ | 348 | 19.3% | 348 | 15.2% | 696 | 17.2% | 1.405 | -16.034^‡^ |
| Mwanza | 20 | 65.0% | 16 | 50.0% | 36 | 58.3% | 0.907 | 1.567 | 48 | 64.6% | 48 | 43.8% | 96 | 54.2% | 2.048^†^ | 2.747^†^ |
| Neno | 75 | 37.3% | 60 | 40.0% | 135 | 38.5% | -0.316 | -1.656 | 180 | 40.0% | 180 | 36.1% | 360 | 38.1% | 0.760 | -0.838 |
| Nsanje | 110 | 64.5% | 88 | 47.7% | 198 | 57.1% | 2.376^†^ | 3.302^†^ | 264 | 41.7% | 264 | 31.4% | 528 | 36.6% | 2.440^†^ | -1.740^†^ |
| Thyolo | 160 | 40.6% | 128 | 5.5% | 288 | 25.0% | 6.847^‡^ | -8.017^‡^ | 384 | 22.4% | 384 | 7.8% | 768 | 15.1% | 5.643^‡^ | -19.422^‡^ |
| National Total | 3,025 | 46.2% | 2,420 | 44.5% | 5,445 | 45.5% | 1.315 |  | 7,260 | 43.3% | 7,260 | 37.1% | 14,520 | 40.2% | 7.667^‡^ |  |

Z-Score 1: Difference of report timeliness between dry season and rainy season within the national, zonal and district strata with two-tailed hypothesis (α=0.05, Z ≥ 1.960 or Z ≤ -1.960)

Z-Score 2: Difference of report timeliness between zone/district annual performance and the national average with one-tailed hypothesis (α=0.05, Z ≥ 1.645 or Z ≤ -1.645)

^*^Studied district  ^†^ P-value < 0.05 ^‡^ P-value < 0.0001
