# Supplementary material for: Integrated Disease Surveillance and Response (IDSR) in Malawi: Implementation gaps and challenges for timely alert
Source: PLoS One. 2018 Nov 29;13(11):e0200858. doi: 10.1371/journal.pone.0200858 (PMC6264833; doi:10.1371/journal.pone.0200858)
Supplement: S5 Table — (DOCX) [file pone.0200858.s005.docx]

**S5 Table. The Malawi IDSR core functions and activities at each health system level comparing between guideline and practice**

| **Level of Health System** | **Case Identification Function** | | **Reporting Function** | |
| --- | --- | --- | --- | --- |
|  | **IDSR Technical Guideline** | **Actual Practice** | **IDSR Technical Guideline** | **Actual Practice** |
| **Community** | - Use simple case definitions to identify priority diseases, public health events, conditions or other hazards in the community | - Case definitions were minimum applied and mainly targeting children - HSAs are not doing actual case finding, instead the volunteers from the village health committees (VHC) are doing the task | - Report case critical information on priority diseases, public health events, conditions, or hazards to health facility and appropriate authorities | - Compile IDSR reports for the attached health facilities - Report critical information when volunteers from VHC inform the HSAs - HSAs perform patient referral for both children and adults - Manual reporting without using standardized tool - Reporting more based on individual health programmes - Not performing weekly IDSR reporting but monthly |
| **Health Facility** | - Use standard case definitions to detect confirm and record priority diseases or conditions public health events - Collect and transport specimens for laboratory confirmation - Use local laboratory capacity to confirm cases or to initiate confirmation of cases if possible | - Standard case definitions were applied - Priority diseases specimens were collected for laboratory confirmation - Minimum laboratory confirmations were done | - Report case-based information for immediately notable diseases - Report summary data to next level - Report laboratory results from screening of sentinel populations - Report laboratory results to next level | - Paper registers as the main tools for data collection and reporting - Mixed with computer system to compile required reports - Not performing weekly report but monthly |
| **District, Zonal** | - Collect surveillance data from reporting sites including designated points of entry on time and review the quality - Ensure reliable supply of data collection and reporting tools are available at reporting sites - Collect and transport specimen for laboratory confirmation - Use local laboratory capacity to confirm cases if possible | - Surveillance data was collected but the quality is poor - Updated IDSR technical guidelines and tools were ready for distribution but not yet performed - Priority diseases specimens were collected for laboratory confirmation - Minimum laboratory confirmations were done | - Make sure health facilities know and use standard case definitions for reporting priority diseases, conditions and public health events - Make sure health facility staff know when and how to report priority diseases, conditions and public health events - Report data on time to the next level - Report laboratory results to next level - Periodically conduct risk assessment for priority diseases, public health events, condition or hazards | - Collecting monthly IDSR reports from facilities - Inadequate filed supervision and trainings to provide to health facility staff - IDSR reports were collected but facing difficulties to submit on time - Lab results are submitted to the next level according irregularly - Irregularly conduct risk assessment |
| **National** | - Define, update and ensure compliance with national policy and guidelines - Set policies and procedures for the national laboratory networks including quality assurance systems - Use national laboratory for confirmatory and specialized testing if necessary - Collect and transport specimens for additional analysis at WHO Collaborating Centres as necessary | - National guideline was updated in 2015 - Lab policies, procedures and networks are in place but not well functioning | - Report the immediate notifiable diseases and public health events to the appropriate authorities on time - Report other priority disease and public health events on time - Include all relevant laboratories in the reporting network - Use IHR Decision Instrument to determine risks for priority diseases public health events, conditions or hazards - Inform WHO as indicated by IHR (2005) | - Reporting immediate notifiable diseases and public health events to WHO, CDC, UNICEF and relevant key stakeholders - Lab information are available but not in good quality (completeness and timeliness) |
