# Supplementary material for: Integrated Disease Surveillance and Response (IDSR) in Malawi: Implementation gaps and challenges for timely alert
Source: PLoS One. 2018 Nov 29;13(11):e0200858. doi: 10.1371/journal.pone.0200858 (PMC6264833; doi:10.1371/journal.pone.0200858)
Supplement: S1 File — (DOCX) [file pone.0200858.s006.docx]

Appendix 1.English Interview questionnaires and observation guideline to understand the surveillance and reporting system of ARIs in Malawi

| **Name of Interviewer:** |  | Interview date: | dd/mm/yyyy |
| --- | --- | --- | --- |
| Interview time: | __hh_:_mm_~_hh_:_mm__ | Attended by: |  |
|  |  |  |  |
| **Code of Interviewee:** |  | Position: |  |
| Duration of the post: | ______years, _______months | Years of civil services: |  |
| Highest Education |  | Year of obtained |  |

**Questions:**

1. What does ‘acute respiratory infection’ mean to you?
2. Can you explain how do you get the ARIs reports?
3. What kind of challenges do you have to get ARIs reports?
4. What will you do when you receive the ARIs reports?
5. What are your opinions about the current ARIs reporting system, and why?
6. How could the ARIs report be improved in Malawi?

**Observations:**

1. May I have a look at the reports you have with regards to ARIs?
2. Observe the way of report archiving.
3. Observe the if the offices display the ARIs reports or trends on boards.

Appendix 2.English Interview guideline to understand communities’ knowledge on ARIs

| **Name of Interviewer:** |  | Interview date: | dd/mm/yyyy |
| --- | --- | --- | --- |
| Interview time: | __hh_:_mm_~_hh_:_mm__ | Attended by: |  |
|  |  |  |  |
| **Code of Interviewee:** |  | Village: |  |
| Gender: | ⃞Male ⃞Female | Age: |  |
| Social Group: | ⃞villager ⃞ |  |  |

**Questions:**

1. What does it mean ‘acute respiratory infection’ to you?
2. Have you ever have acute respiratory infection experience before?
3. What will you do when you have acute respiratory infection illness?
4. What types of care will you do when someone has ARIs?
5. What makes you decide to go to health facility for ARI treatment?

Appendix 3.English Interview and observation guideline for the health care workers on referral procedures of ARIs patient

| **Name of Interviewer:** |  | Interview date: | dd/mm/yyyy |
| --- | --- | --- | --- |
| Interview time: | __hh_:_mm_~_hh_:_mm__ | Attended by: |  |
|  |  |  |  |
| **Code of Interviewee:** |  | Name of Health Facility: |  |
| Gender: | ⃞Male ⃞Female | Age: |  |
| Job Category: |  |  |  |
| Highest Education: |  | Year of obtained |  |

**Questions:**

1. How do you recognize an individual with ARIs?
2. What are the challenges for you to make differential diagnosis to the ARIs patient?
3. At what stage will you decide to referral the patient to the next level?
4. What will make you alert that you have increased/abnormal cases of ARIs in your catchment are?
5. What measures do you take when you are treating ARIs patients for yourself and other people?

**Observation:**

1. Observe the clients who attended by the interviewee and record the signs of symptoms, ways of interviewee making diagnosis and treatment. (Both adult and pediatric patients; adherence to guidelines, check-up procedures, etc.)
2. Observe the equipment for interviewee to do medical checkup and diagnosis.
3. Observe the working environment of the interviewee and see if there is any ARIs related treatment guideline posted or placed, protection units, nosocomial infection prevention materials.

Appendix 4.English Interview guide and case scenario for health care workers to make differeintial diagnosis and express challenges for differentiating:

1. Interview guide:
   1. The interviewers will start the interview with self-introduction and clearly explain the purpose of the study and inform interviewees that the conversation will be recorded.
   2. After obtain the consent from the interviewee, interviewer shall switch on the tape recorder and start the interview.
   3. The interviewer shall follow the semi-structure questionnaire to provoke interviewees’ knowledge toward the research topic.
   4. The interviewer shall keep the pace of interview and control it within suitable timeframe.
2. Hypothetical case scenarios:
   1. If a patient come with symptoms of ‘sneezing, runny nose, cough, sore throat, stuffed nose’, what kind of diagnosis will you give to the patient?
   2. If a patient come with symptoms of ‘sneezing, runny nose, cough, sore throat, stuffed nose, fever, muscle pain, weakness’, what kind of diagnosis will you give to the patient?
   3. If a patient comes with symptoms of ‘cough, fever, muscle pain, weakness, short of breathing’, what kind of diagnosis will you give to the patient?
   4. If a patient come with symptoms of ‘runny nose, sneezing, watery eyes, stuffed nose’, what kind of diagnosis will you give to the patient?
   5. Malaria and other diseases scenarios
3. Reviewing these cases, what are the challenges for you to make differential diagnosis?
