# Supplementary material for: Integrated Disease Surveillance and Response (IDSR) in Malawi: Implementation gaps and challenges for timely alert
Source: PLoS One. 2018 Nov 29;13(11):e0200858. doi: 10.1371/journal.pone.0200858 (PMC6264833; doi:10.1371/journal.pone.0200858)
Supplement: S2 File — (DOCX) [file pone.0200858.s007.docx]

Appendix 1.Chewa Mafunso a muKafukufuku komanso Ndondomeko ya Kauni Ofuna Kuyang’anira mwachidwi momwe zinthu zimagwiritsidwira ntchito komanso Ndondomeko ya Katumizidwe ka Zotsatira za Matenda okhudza Mapumidwe ofika pachimake mu Malawi

| **Dzina la Ofunsa Mafunso amukafukufuku:** |  | Tsiku lofunsira mafunso amukafukufuku: | tsiku/mwezi/chaka |
| --- | --- | --- | --- |
| Nthawi yofunsira mafunso amukafukufuku: | __maola_:_mphindi_~_maola_:_mphindi__ | Kuthandizidwa Ndi: |  |
|  |  |  |  |
| **Nambala yoyimira Ofunsidwa Mafunso:** |  | Udindo: |  |
| Kutalika kwa nyengo yomwe mwakhalira pa udindowu: | ______Zaka, _______miyezi | Zaka zogwilira ntchito muboma: |  |
| Malekezero a Maphunziro: |  | Chaka chomwe mudalandilira zotsatira za maphunziro: |  |

**Mafunso:**

1. Kodi ‘matenda okhudza mapumidwe ofika pachimake’ akutanthauza chiyani kwa inuyo?
2. Mungafotokozeko momwe mumatengera mauthenga a zotsatira za matendawa?
3. Kodi mumakumana ndi mavuto otani potenga zotsatira za matendawa?
4. Kodi mumapanga chiyani mukalandira zotsatirazi?
5. Kodi maganizo anu ndi otani pa zandondomeko yotengera zotsatirazi yomwe ikugwiritsidwa ntchito panopa? Ndipo Chifukwa chiyani?
6. Kodi uthenga wa zotsatira za matenda okhudza mapumidwe zingakonzedwe bwanji kuti zipite patsogolo muMalawi?

**Kauni:**

1. Ndingaoneko nawo zotsatira zomwe muli nazo zokhudza matendawa?
2. Onetsetsani njira yomwe amasungira zotsatirazi
3. Onaninso ngati ogwira ntchito mumaofesiwa amawonetsa mauthenga a zotsatira zamatendawa kapena chiwerengero cha odwala matendawa kwa anthu omwe amayang’anira za matendawa.

Appendix 2.Chewa Ndondomeko ya mafunso a muKafukufuku pofuna kumvetsetsa zomwe anthu a muMudzi akudziwapo pa matenda okhudza mapumidwe ofika pachimake.

| **Dzina la Ofunsa Mafunso a muKafukufuku:** |  | Tsiku lofunsira mafunso a muKafukufuku: | tsiku/mwezi/chaka |
| --- | --- | --- | --- |
| Nthawi yofunsira mafunso a muKafukufuku: | __maola_:_mphindi_~_maola_:_mphindi__ | Kuthandizidwa ndi: |  |
|  |  |  |  |
| **Nambala yoyimilira ofunsidwa mafunso:** |  | Mudzi: |  |
| Mamuna/Mkazi: | ⃞Mamuna ⃞Mkazi | Zaka: |  |
| Social Group: | ⃞Mzika ya mmudzi ⃞ |  |  |

**Mafunso:**

1. Kodi ‘matenda okhudza mapumidwe ofika pachimake’ akutanthauza chiyani kwa inuyo?
2. Kodi munadwalapo kapena kukumana ndi vuto la munthu wodwala nthenda imeneyi?
3. Kodi munapangapo (kapena mumapanga) chiyani mutadwala kapena mukakumana ndi munthu wodwala matendawa?
4. Kodi chithandizo chomwe mumapereka ndi chotani munthu akadwala matendawa?
5. Kodi chomwe chimakupangitsani ndi chiyani kupita kuchipatala kukalandira chithandizo cha matendawa?

Appendix 3.Chewa Ndondomeko ya Mafunso a muKafukufuku komanso ya Kauni yogwiritsira ntchito pofunsa ogwira ntchito muchipatala pa ndondomeko yotumizira odwala matenda okhudza mapumidwe ofika pachimake

| **Dzina la Wofunsa Mafunso a muKafukufuku:** |  | Tsiku lofunsira mafunso a muKafukufuku: | tsiku/mwezi/chaka |
| --- | --- | --- | --- |
| Nthawi yofunsira mafunso a muKafukufuku: | __maola_:_mphindi_~_maola_:_mphindi__ | Kuthandizidwa ndi: |  |
|  |  |  |  |
| **Nambala ya wofunsidwa mafunso a muKafukufuku:** |  | Dzina la Chipatala: |  |
| Mamuna/Mkazi: | ⃞Mamuna ⃞Mkazi | Zaka: |  |
| Gulu la Ntchito yanu: |  |  |  |
| Malekezero a Maphunziro: |  | Chaka chomwe mudalandilira zotsatira za maphunziro: |  |

**Mafunso:**

1. Kodi mumamuzindikira bwanji munthu yemwe wadwala matenda okhudza amapumidwe?
2. Kodi mavuto omwe mumakumana nawo poyeza vuto lenileni la munthu wodwala matendawa ndi ati?
3. Kodi mumamutumiza wodwala matendawa kuchipatala chachikulu matenda akafika pokula bwanji?
4. Kodi chimene chingakudziwitseni kuti chiwerengero cha anthu omwe adwala matendawa chakula ndi chiyani?
5. Kodi mumatsatira ndondomeko ziti mukamawathandiza anthu odwala matendawa?

**Kauni:**

1. Onani mwachidwi anthu odwala omwe adathandizidwa ndi wofunsidwa mafunso a muKafukufukuyu ndipo mulembe zizindikiro za odwala matendawa zomwe adalemba, njira zimene adagwiritsa ntchito powayeza odwalawa pofuna kudziwa vuto lenileni komanso chithandizo chimene chidaperekedwa kwa odwalawa. (Onani za Odwala aakuru komanso ana; katsatidwe ka ndondomeko zomwe zidakhazikitsidwa, ndondomeko zowazondera odwala matendawa, ndi zina)
2. Onaninso zipangizo zomwe amene akuyankha mafunso amuKafukufukuyu amagwiritsa ntchito pothandizira odwala matendawa.
3. Onaninso malo omwe amagwilira ntchito amene akufunsidwa mafunsowa komanso yang’anani ngati ndondomeko zilizonse zokhudzana ndi kuthandiza odwala matendawa, zipangizo zodzitetezera ku ngozi, komanso ngati zipangizo zoteteza ku matenda a mapumidwe omwe amatha kupatsirana zinakhomedwa kapena zinapachikidwa pa malo pena pake.

Appendix 4.Chewa Ndondomekoya mafunso a muKafukufuku komanso ena mwa madandaulo omwe odwala matenda okhudza mapumidwe ofika pachimake angathe kubwera nawo kwa ogwira ntchito pa chipatala kuti angawayeze bwanji odwalawa komanso ndi mavuto anji omwe amakumana nawo pofuna kupeza vuto lenileni la odwala matendawa:

1. Ndondomeko ya mafunso:
   1. Wofunsa mafunso adzayamba kufunsa mafunso a muKafukufukuyu pozidziwitsa yekha kwa ofunsidwa mafunso, komanso adzafotokoza tsatanetsatane wa cholinga cha kafukufukuyu komanso kuwadziwitsa ofunsidwa mafunsowa kuti macheza awa awasunga mu chipangizo chotapira mawu.
   2. Akadzatha kulandira chilorezo chopitiriza kufunsa mafunso, ofunsa adzayatse chipangizo chotapira mawu ndipo ayambe macheza.
   3. Ofunsa atsatire mafunso omwe akhazikitsidwa muKafukufukuyu opangitsa kuti munthu oyankha adzifotokoza zambiri zokhudza mutu wa kafukufuku
   4. Ofunsa asunge nthawi yofunsira komanso kuyankhira mafunso a muKafukufuku mu nyengo yomwe yakhazikitsidwa.
2. Zitsanzo za momwe odwala matendawa angathe kuanena komanso angakhale nazo:
   1. Ngati wodwala wabwera ndi zizindikiro zofwenthera, chimfine, kutsokomola, zilonda za pakhosi ndi mphuno zotseka, Kodi mungamuyeze zotani wodwalayu?
   2. Ngati wodwala wabwera ndi zizindikiro zofwenthera, chimfine, kutsokomola, zilonda za pakhosi, kutentha thupi, kumva kupweteka muminyewa komanso kufooka kwa thupi, kodi mungamuyeze zotani wodwalayu?
   3. Ngati wodwala wabwera ndi zizindikiro zotsokomola, kutentha thupi, kumva kupweteka muminyewa, kufooka kwa thupi komanso kupuma mopelewera, mungamuyeze zotani wodwalayu?
   4. Ngati wodwala wabwera ndi zizindikiro za chimfine, kufwenthera, kumangotuluka misonzi mmaso komanso mphuno zotseka.
   5. Malungo komanso zitsanzo za matenda ena
3. Mutaona zitsanzo za kabweredwe ka matenda zili mu mwambamu, kodi mavuto amene mumakumana nawo kuti mupeze vuto lenileni la matenda ndi otani?
