# Supplementary material for: Integrated Disease Surveillance and Response (IDSR) in Malawi: Implementation gaps and challenges for timely alert
Source: PLoS One. 2018 Nov 29;13(11):e0200858. doi: 10.1371/journal.pone.0200858 (PMC6264833; doi:10.1371/journal.pone.0200858)
Supplement: S3 File — (DOCX) [file pone.0200858.s008.docx]

Appendix 1.Tumbuka Mafumbo gha muKafukufuku na ndondomeko ya kalabiskiro ka umo ntchito ikugwirikira na umo ndondomeko ya katumiro ka vyakusatira vya matenda bakukhwaska kathutiro muMalawi.

| **Zina la wakufumba mafumbo gha muKafukufuku** |  | Dazi la kufumbira mafumbo gha muKafukufuku | dazi/mwezi/chaka |
| --- | --- | --- | --- |
| Nyengo ya kufumbira mafumbo gha muKafukufuku | __maola_:_mphindi_~_maola_:_mphindi__ | Kuvwirika na: |  |
|  |  |  |  |
| **Nambala yakuyimilira uyo wakuzgola mafumbo:** |  | Udindo: |  |
| Nyengo iyo mwakhalirapo pa udindo ughu: | ______vyaka, _______miyezi | Vyaka ivo mwagwilira ntchito mu boma: |  |
| Apo muli kufikira na masambiro: |  | Chaka icho mukapokera vakulata vya masambiro ghinu: |  |

**Mafunso:**

1. Kasi ‘matenda gha kukhwaska kathutiro’ ghakung’anamula vichi kwa imwe?
2. Munganiphalirako umo mukutolera vakulata vya matenda agha?
3. Kasi mukusangana na masuzgo ngani pala mukutola vakulata vya matenda agha?
4. Kasi pala mwapokera vakulata ivi mukuchita vichi?
5. Kasi maghanoghano ghinu ni ngani pa ndondomeko iyo ikugwira ntchito sono yakutolera vakulata ivi?
6. Kasi vilato vya matenda agha zinganozgeka uli kuti vilute panthazi muMalawi?

**Kalabiskiro:**

1. Ningabekako vakulata ivo mungaba navo vyakukhwaska matenda agha?
2. Bekeskani nthowa iyo bakugwiriska ntchito pakusunga vakulata ivi.
3. Labiskaniso pala abo bakugwira ntchito mumaofesi agha bakulongola mauthenga ba vakulata ivi ku banthu abo bali balalabalala ba matenda agha.

Appendix 2.Tumbuka Ndondomeko ya mafumbo gha muKafukufuku bakukhumba kupulikiska ivo banthu bamuzikaya bakumanyapo pa matenda gha kukhwaska kathutiro.

| **Zina la wakufumba mafumbo gha muKafukufuku** |  | Dazi la kufumbira mafumbo gha muKafukufuku: | dazi/mwezi/chaka |
| --- | --- | --- | --- |
| Nyengo yakufumbira mafumbo gha muKafukufuku: | __maola_:_mphindi_~_maola_:_mphindi__ | Kuvwirika na: |  |
|  |  |  |  |
| **Nambala yakuyimilira uyo wakuzgola mafumbo:** |  | Kukaya: |  |
| Ubiro winu: | ⃞Mwanalume ⃞Mwanakazi | Vyaka: |  |
| Social Group: | ⃞Mzika wa mukaya ⃞ |  |  |

**Mafumbo:**

1. Kasi ‘matenda gha kukhwaska kathutiro’ ghakung’anamula vichi kwa imwe?
2. Kasi muli kulwarapo panji kusangana na munthu uyo wali na matenda agha?
3. Kasi mukachita vichi apo mukalwara panji apo mukasangana na munthu uyo wakalwara matenda agha?
4. Kasi wovwiri uwo mukovwira ni wakuti uli pala munthu walwara matenda agha?
5. Kasi icho chikumukanikisgani kuluta ku chipatala kukapokera wovwiri wa matenda agha ni vichi?

Appendix 3.Tumbuka Ndondomeko ya Mafumbo gha muKafukufuku na chilabiskiro chakugwiriska ntchito pala bakugwira ntchito muchipatala bakufumbika mafumbo gha muKafukufuku ughu na ndondomeko yakutumira baluwari ba matenda agha kuchipatala chikulu

| **Zina la wakufumba mafumbo gha muKafukufuku:** |  | Dazi la kufumbira mafumbo gha muKafukufuku: | dazi/mwezi/chaka |
| --- | --- | --- | --- |
| Nyengo yakufumbira mafumbo gha muKafukufuku: | __maola_:_mphindi_~_maola_:_mphindi__ | Kuvwirika na: |  |
|  |  |  |  |
| **Nambala yakuyimilira uyo wakuzgola mafumbo:** |  | Zina la Chipatala: |  |
| Ubiro winu: | ⃞Mwanalume ⃞Mwanakazi | Vyaka: |  |
| Gulu la Ntchito yinu: |  |  |  |
| Apo muli kufikira na masambiro: |  | Chaka icho mukapokera vakulata vya masambiro ghinu: |  |

**Mafunso:**

1. Kasi mukumuzindikira uli munthu uyo waluwara matenda gha kukhwaska kathutiro?
2. Kasi masuzgo abo mukusangana nawo pala mukupima suzgo lenecho la abo baluwara matenda agha ni vichi?
3. Kasi mukumutuma muluwari wa matenda agha kuchipatala chikulu pala matenda ghali mbuni?
4. Kasi icho chingamumanyiskani kuti nambala ya banthu awo baluwara matenda agha yakula ni vichi?
5. Kasi pala mukuvwira baluwari mukugwiriska ntchito ndondomeko zini?

**Kalabiskiro:**

1. Labiskani chomene banthu baluwari awo bali kuvwirikapo na uyo wakuzgola mafumbo ndipo mulembe zizindikiro vya labuwari ba matenda agha ivo vili kulembeka, nthowa iyo wakagwiriska ntchito apo wakawapimanga baluwari kuti wasange suzgo lenecho na wovwiri uwo ukaperekeka kwa waluwari agha. (Labiskani vya baluwari balala na bana; kagwiliro kantchito ka ndondomeko izo zili kubikika, ndondomeko zakubaonera baluwari agha pala bapokera wovwiri, na vinyakhe)
2. Labiskaniso viyawiro ivo abo bakuzgola mafumbo gha muKafukufuku bakugwiriska ntchito pala bakuvwira baluwari.
3. Labiskaniso malo abo bakugwilirapo ntchito abo bakuzgola mafumbo ndipo mulabiskeso pala vinthu ivi vilipo; ndondomeko zilizose zakukhwaskana na kovwira baluwari ba matenda agha, viyawiro vyakujithaskira ku ngozi, na viyawiro zakujithaskira ku matenda gha mathutiro agho ghangamanya kutoleka kufuma kwa munthu munyakhe, vili kupayikika pakhoma panji pamalo ghanyakhe ghakuti wali yose wangawona.

Appendix 4.Tumbuka Ndondomeko ya mafumbo gha muKafukufuku na madando anyakhe abo baluwari ba matenda akukhwaskana na kuthuta ghangafika nabo kuchipatala nakuti bangapimika uli na masuzgo abo bakugwira ntchito muchipatala bangasangana nawo pala bakukhumba kusanga suzgo lenecho ka baluwari agha:

1. Ndondomeko ya mafumbo:
   1. Uyo wakufumba mafumbo wadankhe na kufumba mafumbo gha mukafukufuku ughu pakujimanyiksa yekha kwa uyo wazgolenge mafumbo, kusazgira apo, wafotokoze chilato cha kafukufuku ughu na kuwamanyiska kuti kuchezga uku basungenge mu chiyawiro icho bakutapira mazgo.
   2. Pala wapokera chizomerezgo chakulutilira kufumba mafumbo, uyo wakufumba mafumbo wabuske chiyawiro chakutapira mazgo ndipo bayambepo kuchezga.
   3. Uyo wakufumba mafumbo walondozge mafumbo abo banozgekera mukafukufuku ughu abo bakuchitiska munthu kuyowoya vinandi cyakukhwaska mutu wa kafukufuku.
   4. Uyo wakufumba mafumbo wakole nyengo yakufumbira mafumbo na kuzgolera mafumbo gha mukafukufuku munyengo iyo ili kuyikika.
2. Viyerezgero vya umo baluwari ba matenda agha bangayowoya panji bangiza navo kuchipatala:
   1. Pala muluwari wiza na vimanyisko vya kufwenkhula, chimfine, chikhoso, vilonda vya pasingo na mphuno zakujala, kasi mungamupima uli muluwari uyu?
   2. Pala muluwari wiza na vimanyisko vya kufwenkhula, chimfine, chikhoso, vilonda vya pa singo, kuotcha thupi, kupulika kubinya mu nthinthi na kuzotofyeka kwa thupi, kasi mungamupima vichi muluwari uyu?
   3. Pala muluwari wiza na vimanyisko vya chikhoso, kuotcha thupi, kupulika kubinya mu nthinthi, kuzotofyeka kwa thupi na kusuzgikira kuthuta, mungamupima vichi muluwari uyu?
   4. Pala muluwari wiza na vimanyisko vya chimfine, kufwenkhula, kufuma masozi mumaso na mphuno zakujara, mungamupima vichi muluwari uyu?
   5. Malungo na viyerezgero vya matenda ghanyakhe.
3. Pala mwamala kuona visazo vya umo matenda ghali muchanya bakwizira, kasi masuzgo agho mukusangana nabo kuti musange suzgo lenecho la muluwari ni vichi?
